# Does choice of outdoor heat metric affect heat-related epidemiologic analyses in the US Medicare population?

**DOI:** 10.1097/EE9.0000000000000261

**Published:** 2023-07-13

**Authors:** Keith R. Spangler, Quinn H. Adams, Jie Kate Hu, Danielle Braun, Kate R. Weinberger, Francesca Dominici, Gregory A. Wellenius

**Affiliations:** a Department of Environmental Health, Boston University School of Public Health, Boston, Massachusetts; b Department of Biostatistics, Harvard University T.H. Chan School of Public Health, Boston, Massachusetts; c Department of Data Sciences, Dana-Farber Cancer Institute, Boston, Massachusetts; d University of British Columbia, School of Population and Public Health, Vancouver, British Columbia, Canada

## Abstract

Outdoor air temperature is associated with increased morbidity and mortality. Other thermal indices theoretically confer greater physiological relevance by incorporating additional meteorological variables. However, the optimal metric for predicting excess deaths or hospitalizations owing to extreme heat among US Medicare beneficiaries remains unknown.

**Methods:**

We calculated daily maximum, minimum, and mean outdoor air temperature (T), heat index (HI), wet-bulb globe temperature (WBGT), and Universal Thermal Climate Index (UTCI) for populous US counties and linked estimates with daily all-cause mortality and heat-related hospitalizations among Medicare beneficiaries (2006–2016). We fit distributed-lag nonlinear models for each metric and compared relative risks (RRs) at the 99th percentile.

**Results:**

Across all heat metrics, extreme heat was statistically significantly associated with elevated risks of morbidity and mortality. Associations were more pronounced for maximum daily values versus the corresponding minimum for the same metric. The starkest example was between HI_max_ (RR = 1.14; 95% confidence interval [CI] = 1.12, 1.15) and HI_min_ (RR = 1.10; 95% CI = 1.09, 1.11) for hospitalizations. When comparing RRs across heat metrics, we found no statistically significant differences within the minimum and maximum heat values (i.e., no significant differences between T_max_/HI_max_/WBGT_max_/UTCI_max_ or between T_min_/HI_min_/WBGT_min_/UTCI_min_). We found similar relationships across the National Climate Assessment regions.

**Conclusion:**

Among Medicare beneficiaries in populous US counties, daily maximum and mean values of outdoor heat are associated with greater RRs of heat-related morbidity and all-cause mortality versus minimum values of the same metric. The choice of heat metric (e.g., temperature versus HI) does not appear to substantively affect risk calculations in this population.

**What this study adds**

There is considerable interest in measures of outdoor heat that combine multiple weather variables to approximate how “hot” it feels to the human body (e.g., wet-bulb globe temperature). However, few studies have systematically determined whether these complex measures are more associated with the health impacts of heat at the population scale. Our study compares different ways of measuring heat and provides evidence that all hot days—regardless of how “hot” is measured—are associated with similar levels of excess risk of death or heat-related hospitalization in the US Medicare population.

## Introduction

Moderate and extreme heat have been extensively documented as being associated with a higher risk of death or hospitalization.^1^ Most heat-related epidemiologic studies use either outdoor dry-bulb temperature (the standard measurement of air temperature) or heat index (HI) (an algorithmic composite of air temperature (T) and relative humidity) as the exposure metric (e.g.,^2–6^), but other existing thermal comfort indices could theoretically better approximate human physiological responses to heat. Two popular metrics of this sort include the wet-bulb globe temperature (WBGT)^7,8^ and the more recently developed Universal Thermal Climate Index (UTCI),^9^ both of which are composite indices that are responsive to air temperature, humidity, wind speed, and solar radiation and are intended to reflect human thermal comfort. In the United States, WBGT has been used operationally for decades in athletic, military, and occupational contexts to regulate outdoor activity patterns, though some have advocated for instead using the newer UTCI metric in some of these settings to better account for heat severity.^10,11^ Although these metrics have demonstrated utility for *in situ* use—for example, using a WBGT monitor on a football field to make decisions about training duration—their usefulness in population-scale epidemiologic analyses has not been systematically evaluated in the US Medicare population.

Others have compared multiple heat metrics in public health analyses in different locations and populations of interest, with differing results. Barnett et al^12^ compared air temperature to two composite measures of temperature and humidity across major United States cities and found that the “best” predictor of all-cause mortality (excluding accidents) varied by location and population of interest. Similarly, Vaneckova et al^13^ assessed a broader suite of heat metrics in Brisbane, Australia, including an approximation of WBGT, and found no statistically significant differences in associations with all-cause mortality. Metzger et al^14^ also found similar effect estimates on mortality between different heat metrics in New York City. Zhang et al^15^ similarly compared several exposure metrics used to trigger heat warning systems and concluded that the metrics thought to be most effective in characterizing heat stress might not be the same metrics that are best at predicting heat-related mortality. In a more recent study, Lee et al^16^ estimated heat-attributable mortality in Switzerland and South Korea using eight different temperature metrics and showed that there is no measure that consistently performs better than others. By contrast, Heo et al^17^ found that the quantification of the impact of heat waves on health increased considerably for most health outcomes when using an approximation of WBGT.

Our analysis is novel in several ways: (1) No analysis to date has compared heat-related health outcomes by heat metric in the US Medicare population, a particularly heat-vulnerable population that includes the vast majority of Americans over 65 years of age; (2) We assess both daily mortality and heat-related hospitalizations, whereas previous analyses typically evaluate only one outcome or the other; (3) We include a comprehensive array of minimum, maximum, and mean daily metrics of several heat variables; (4) Our calculations of WBGT and UTCI are calculated using gold-standard algorithms rather than linear approximations; and (5) Our analysis has wide geographic coverage across the contiguous US (CONUS), spanning several hundred counties that collectively represent over 80% of the total CONUS population.

Our article provides a widely encompassing intercomparison of the morbidity and mortality impacts of a variety of heat measurements among a nearly comprehensive swath of older US adults. We define extreme-heat days by 12 different heat metrics of interest to the epidemiology community, and we then estimate and compare the relative risks (RRs) of all-cause mortality and heat-related hospitalizations among Medicare beneficiaries using time-series regression modeling.

## METHODS

### Exposure metrics

We calculated population-weighted, daily minimum, maximum, and mean outdoor air temperature, heat index, wet-bulb globe temperature, and Universal Thermal Climate Index at the county resolution from 2006 to 2016 for the contiguous United States (CONUS). The input data come from the European Centre for Medium-Range Weather Forecasts Reanalysis version 5 — Land global reanalysis gridded data set with an hourly temporal resolution and spatial resolution of approximately 9 kilometers. The data used are publicly available and described in great detail in Spangler et al.^18^ Briefly, we calculated WBGT following the approach of Liljegren et al,^8^ which uses a validated algorithm for approximating WBGT using established thermodynamic and physical principles of heat transfer; UTCI following the approaches of Di Napoli et al^19^ and Brimicombe et al;^20^ and HI following the approach of the US National Weather Service as implemented by Anderson et al.^4^ Two-meter air temperature is provided directly by European Centre for Medium-Range Weather Forecasts Reanalysis version 5 — Land. We calculated the minimum, maximum, and mean values of WBGT, UTCI, HI, and air temperature at the grid cell level from the 24 hourly data points in each local day, and then calculated the county mean value by weighting each pixel by its share of the county population, using high-resolution population estimates from the Joint Research Centre of the European Commission’s Global Human Settlement Population (GHS-POP) data set.^21^

### Health data

**Figure 1.**
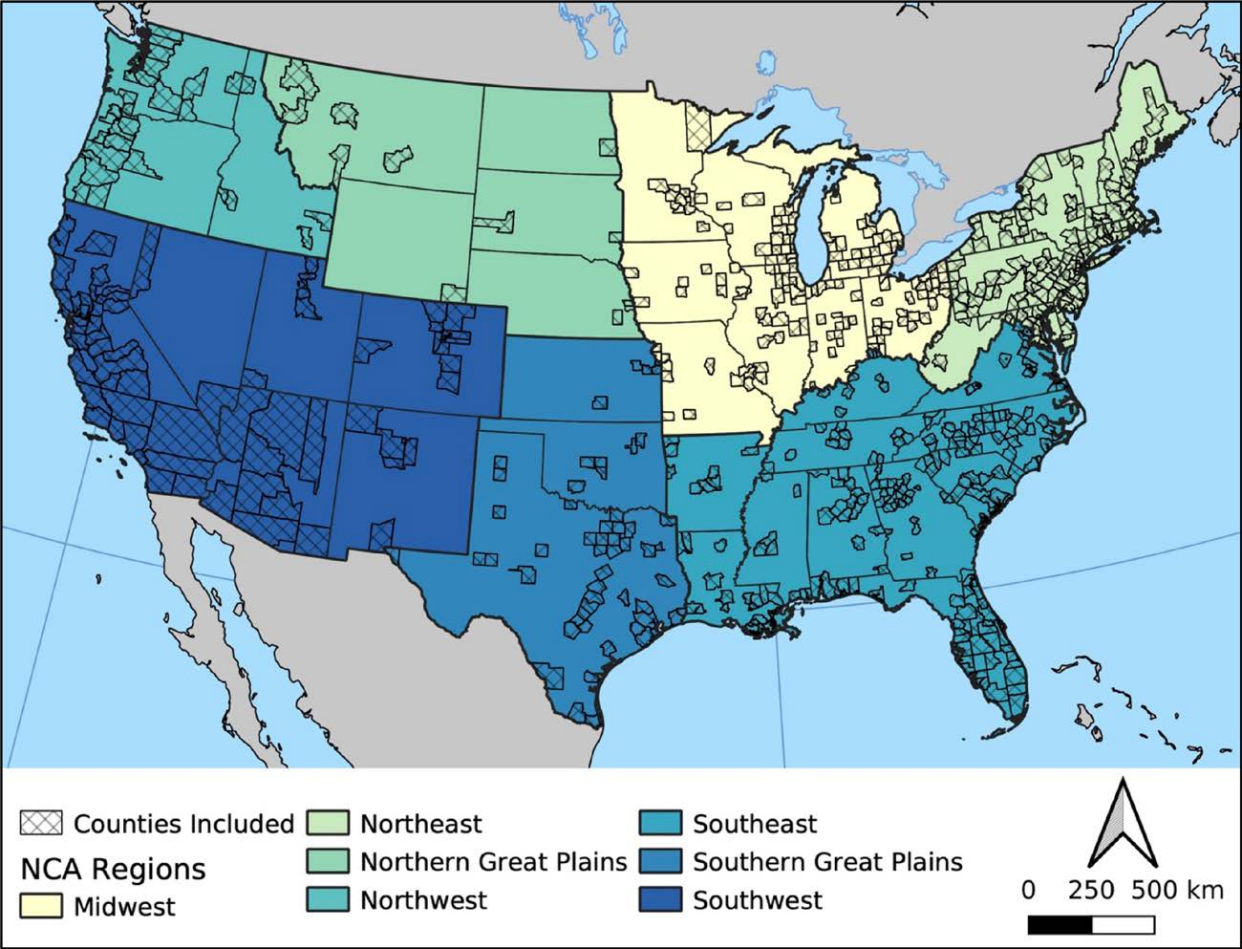
Populous counties included in the analysis (cross-hatched areas) and the National Climate Assessment (NCA) regions (shaded states) in the contiguous United States.

We obtained daily, county-level, all-cause mortality data for the US Medicare population from 2006 to 2016, as well as daily, county-level, cause-specific hospitalizations among Medicare fee-for-service (FFS) beneficiaries over the same period. Medicare is a US government-sponsored health insurance program for individuals at least 65 years old and/or persons with particular disabilities, such as end-stage renal disease.^22^ Our data sets were subsets of individuals aged 65 years or older. We used all-cause mortality counts rather than cause-specific deaths because the latter was not available in our data set. We defined “heat-related hospitalizations” as the total number of hospitalizations among Medicare FFS beneficiaries with Clinical Classifications Software (CCS) codes of 55 (fluid and electrolyte disorders), 157 (acute and unspecified renal failure), 159 (urinary tract infections), 2 (septicemia), 244 (heat and other injuries and conditions owing to external causes), 114 (peripheral and visceral atherosclerosis), and 50 (diabetes mellitus with complications). These CCS codes were assigned from the original International Classification of Disease codes using the *icd* R package,^23^ following the CCS algorithm.^24^ We selected these CCS codes based on recent work on heat morbidity in this population.^25^ While most of these conditions are not directly attributable to heat and therefore can occur under any temperature conditions, prior research has found that they present at statistically significantly higher rates on extreme-heat days and hence are believed to be exacerbated by heat exposure.^25^ We subset our data to populous counties containing at least 80,000 people (as of the 2010 Census) to ensure sufficient sample size and model stability (i.e., including counties with much smaller populations resulted in convergence errors); the included counties comprise 80.6% of the total CONUS population (Figure 1).

### Modeling relative risks of morbidity and mortality

We used time-series regression to separately model the relationships between daily outdoor heat values during the warm season (defined as the five warmest months by daily maximum air temperature for each county, generally May–September) and county-level counts of daily (1) All-cause mortality and (2) Total heat-related hospitalizations (as described above) in the US Medicare population. Specifically, we fit separate distributed-lag nonlinear models (DLNM)^26^ of quasi-Poisson generalized linear models for each heat metric in a two-stage process, following the approach used by Gasparrini et al^27^ for calculating overall warm season heat-health associations, which we summarize here. In the first stage, we fit separate DLNMs for each populous county in CONUS with a natural cubic spline on the day of season interacted with the year (four degrees of freedom for seasonality), a natural cubic spline on the date (one degree of freedom per decade for long-term trends), a cross-basis function, and an indicator variable for day of the week. We specified an internal knot at the 75th percentile of the county-specific heat metric distribution for the exposure-response function, and we specified 5 days of lag, two equally spaced log-value knots, and four degrees of freedom for the lag function. We used 5 days of lag rather than 10 days (as was done in Gasparrini et al^27^) because the health effects in our study population appeared to be contained almost entirely within this time period.

We note that in this time-series modeling framework, only time-varying confounders need to be controlled for in the model;^28^ prevalence of air conditioning, degree of urbanization, and sociodemographic characteristics do not vary appreciably on daily timescales and were therefore not included. Although tropospheric ozone pollution varies on daily timescales and is statistically associated with morbidity and mortality, we did not control for it because ozone is thought to be a causal intermediary in the relationship between heat and morbidity/mortality rather than a confounder.^29^ Our estimates, therefore, reflect the total effect of heat on morbidity/mortality rather than only the direct effect.

In the second stage, we ran a meta-analysis on the estimated county-level coefficients, controlling for both the average and range of warm-season heat metrics specific to each county. To obtain the final nationwide cumulative association, we calculated the predicted pooled coefficients from the meta-regression output, setting the covariates to the average values from all of the included counties. In this step, we centered the predictions at the heat metric minimum-mortality/morbidity percentile (constrained to be between the 10th and 90th percentiles), which was identified using the meta-regression output. We report here the RRs of mortality (for the all-cause mortality models) and heat-related hospitalizations (for the morbidity models) for 99th percentile heat metrics compared with the minimum-mortality/morbidity percentile. We additionally compared RRs at the 95th percentile as a sensitivity analysis. Finally, we repeated this entire process with separate models stratified by counties within each National Climate Assessment (NCA) region (Figure 1). Statistical modeling was performed in R version 4.2.0 using the dlnm (version 2.4.7) and mvmeta (version 1.0.3) packages.^30–32^

### Quantifying concordance in extreme-heat days by metric

Strong correlations at the upper extreme of the heat metrics would result in similar RRs because the different heat metrics would be identifying similar days. To determine the extent to which this could be a factor in our results, we calculated the percentage overlap in extreme-heat days between each combination of two heat metrics. Specifically, for each pair of metrics (e.g., T_max_ and HI_max_), we calculated the proportion of days in which at least one of the heat metrics was in the 99th percentile for the warm season in the period of interest and the other heat metric was at least in the 98th percentile. We allowed for a margin of error under the assumption that heat risks are quite similar between 98th and 99th percentiles. We calculated these concordances nationally and by NCA region.

## RESULTS

### Temporal correlation between extreme-heat days by metric

**Figure 2.**
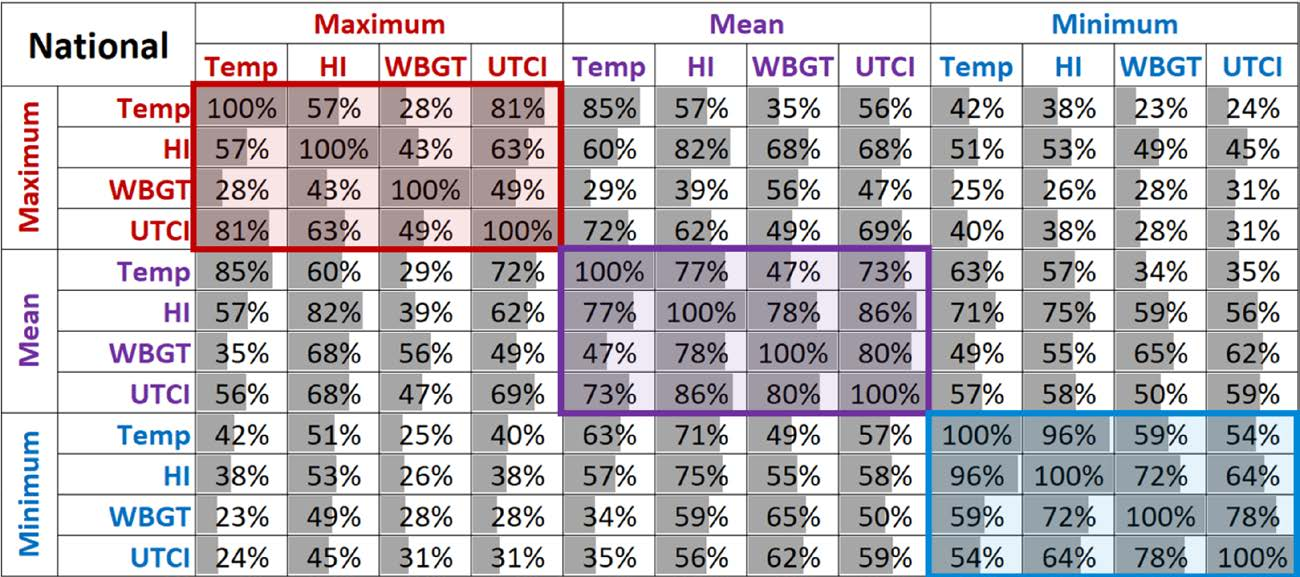
Concordance between extreme-heat days by metric. Values indicate the percentage of days in which the top 1% of hottest days in either of the given metrics were in the top 2% of hottest days by the other metric for the counties included in the analysis over the warm seasons (generally May–September) of 2006–2016. Region-specific concordance tables broadly follow the national patterns and can be found in eFigures S1–S7; http://links.lww.com/EE/A231. HI indicates heat index; temp, air temperature; UTCI, Universal Thermal Climate Index; WBGT, wet-bulb globe temperature.

There was substantial heterogeneity in the particular extreme-heat days (99th percentile) identified by each of the metrics nationally (Figure 2). Among the daily maximum values nationally, the greatest degree of agreement on the hottest days was between T_max_ and UTCI_max_ (81% concordance) and the lowest degree of agreement was between T_max_ and WBGT_max_ (28%). Daily minimum values overall showed greater agreement, particularly between T_min_ and HI_min_ (96%). Daily maxima and minima generally disagreed: the highest concordance was between HI_min_ and HI_max_ (53%), whereas the lowest was between T_max_ and WBGT_min_ (23%). The concordance between daily mean values ranged from 47% between T_mean_ and WBGT_mean_ to 86% between HI_mean_ and UTCI_mean_. These patterns of heat-day concordances generally held within NCA regions (eFigures S1–S7; http://links.lww.com/EE/A231).

### National analysis of health effects

**Figure 3.**
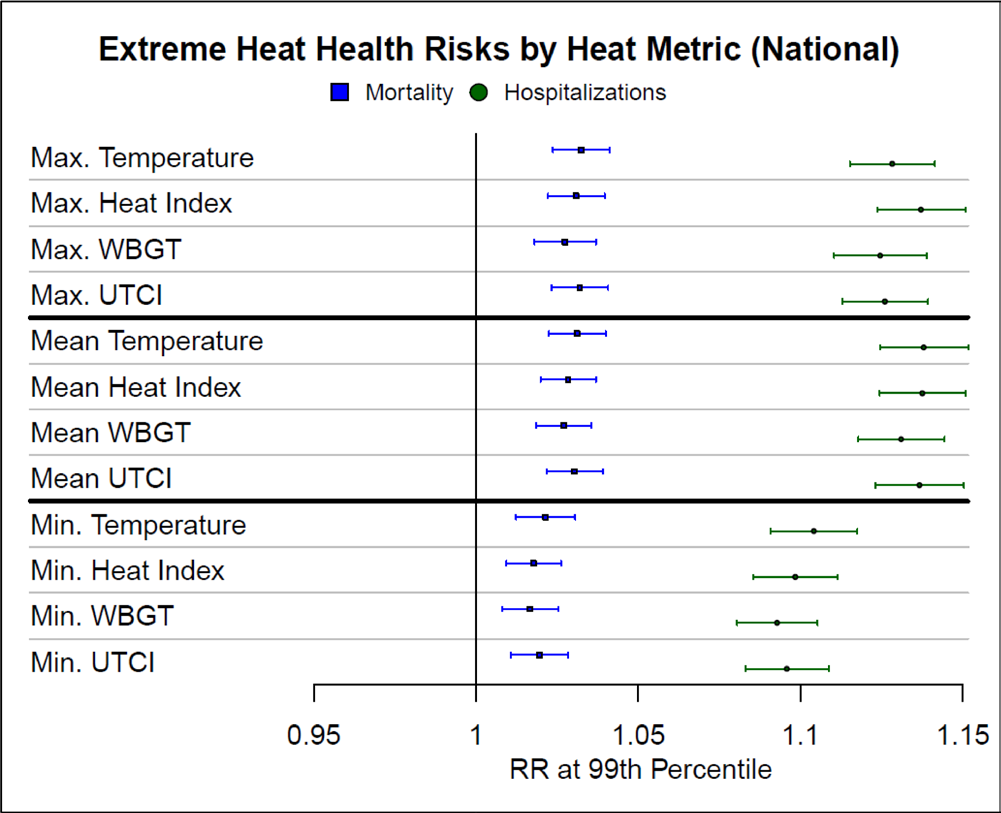
National relative risks (RR) of mortality (blue lines with squares) and heat-related hospitalizations (green lines with circles) at the 99th percentile of different heat metrics in the US Medicare Population, 2006–2016. Relative risks are for the 99^th^ percentile of each heat metric compared to the corresponding heat metric of minimum morbidity/mortality percentile (MMP). Max indicates maximum; min, minimum; UTCI, Universal Thermal Climate Index; WBGT, wet-bulb globe temperature.

The RR of nationwide all-cause mortality and heat-related hospitalizations in the US Medicare population (2006–2016) was statistically significantly elevated at the 99th percentile for all heat metrics assessed (Figure 3). Effect estimates for all-cause mortality were extremely similar across all 12 metrics: all the daily minimum heat values each had an estimate of 1.02 (95% confidence interval [CI] = 1.01, 1.03), and all of the maximum and mean heat values each had an estimate of 1.03 (95% CI = 1.02, 1.04). By contrast, effect estimates for heat-related hospitalizations were consistently larger for the maximum and mean daily values (T_max_, HI_max_, WBGT_max_, and UTCI_max_, as well as T_mean_, HI_mean_, WBGT_mean_, and UTCI_mean_) than the corresponding minimum daily values (T_min_, HI_min_, WBGT_min_, and UTCI_min_). Some, but not all, of these differences were statistically significant. The associations ranged from 1.09 (95% CI = 1.08, 1.11) for WBGT_min_ to 1.14 (95% CI = 1.12, 1.15) for HI_max_. However, there were no statistically significant differences among the different maximum and mean values or among the different minimum values. Finally, we found similar results, albeit with lower RRs, for the 95th percentiles of heat metrics compared with the MMT (eFigure S15; http://links.lww.com/EE/A231).

### Regional analyses of health effects

**Figure 4.**
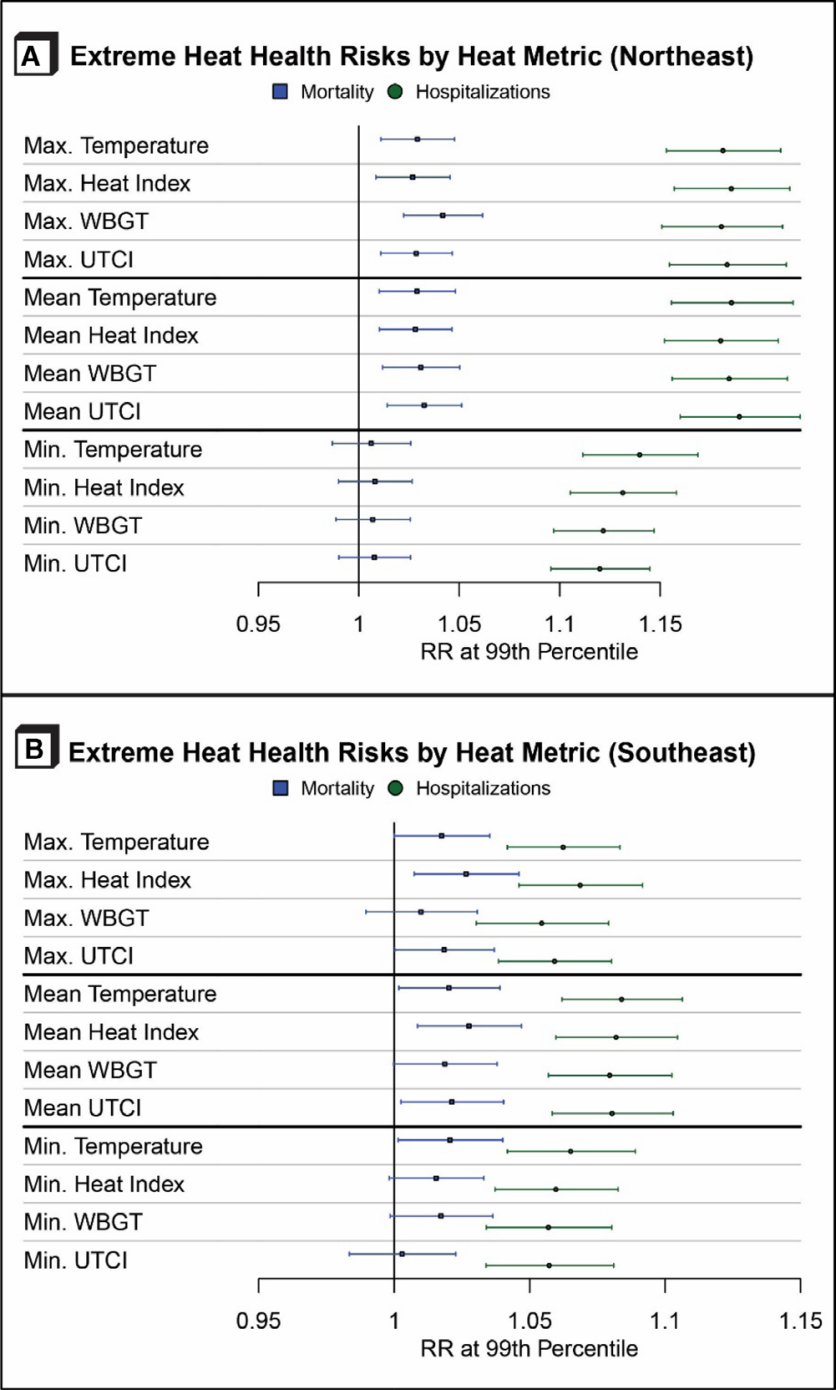
Relative risks (RR) of mortality (blue lines with squares) and heat-related hospitalizations (green lines with circles) at the 99th percentile of different heat metrics in the US Medicare population, 2006–2016, for the Northeast (A) and Southeast (B). Relative risks are for the 99^th^ percentile of each heat metric compared to the corresponding heat metric of minimum morbidity/mortality percentile (MMP). Max indicates maximum; min, minimum.

Analyses stratified by NCA regions generally showed the same patterns in RRs as were seen in the national analysis (eTable S1; http://links.lww.com/EE/A231). In general, all heat metrics provided substantively similar RRs for mortality, whereas the heat-related hospitalization RRs generally were higher for the daily maximum heat metrics than for the daily minimum metrics. In the Northeast, effect estimates for mortality were not statistically significant for any of the daily minimum heat metrics, but all the estimates were significant for the daily maximum metrics (Figure 4A). Differences between minimum and maximum values for hospitalizations were also stark in the Northeast, with RRs ranging from 1.12 (95% CI = 1.10, 1.14) for UTCI_min_ to 1.19 (95% CI = 1.16, 1.21) for HI_max_. In the Northeast, both maximum and mean values for WBGT and UTCI were statistically significantly greater than their counterparts for daily minima. Among all the regions, the risk of heat-related hospitalizations was lowest in the Southeast, with estimates ranging from 1.05 (95% CI = 1.03, 1.08) for WBGT_max_ to 1.07 (95% CI = 1.05, 1.09) for HI_max_. In contrast to other regions, the Southeast had very minimal differences in the effect estimates across any of the heat metrics (Figure 4B). Forest plots for all NCA regions can be found in eFigures S8–S14; http://links.lww.com/EE/A231 (RR at 99th percentile compared to MMT) and in eFigures S16–S22; http://links.lww.com/EE/A231 (RR at 95th percentile).

## DISCUSSION

Our findings are consistent with the consensus in the heat-health literature that extreme heat poses a substantial risk to human health.^1^ Looking specifically at the US Medicare population (for mortality) and the Medicare FFS population (for hospitalizations) in populous CONUS counties, we showed that RRs of all-cause mortality and heat-related hospitalizations are significantly elevated on days of extreme heat, both nationally and for most regions specifically. This concurs with others’ work finding elevated heat-related health risks among older Americans.^25,33–36^

The novelty of our study is in its comparison of multiple heat metrics—including minimum, maximum, and mean wet-bulb globe temperature and the Universal Thermal Climate Index calculated using their respective gold-standard algorithms rather than coarse approximations—for determining both heat-related morbidity and mortality risks for a large portion of the Medicare population, nationally and regionally. Nationally, we found that daily maximum and mean values of most heat metrics result in greater RRs of heat-related hospitalizations than daily minimum values. This dichotomy between minimum and maximum values was found in some—but not all—NCA regions, with the starkest example being in the Northeast, where effect estimates differed by as much as 58%.

Overall, effect estimates were higher in the cooler regions of the northern contiguous United States, consistent with other work finding greater heat-related health effects in cooler climates, both in the US and globally.^37–40^ Although no region showed meaningful differences between the four types of heat metrics for the same daily value (e.g., T_max_ vs. HI_max_ or WBGT_min_ vs. UTCI_min_), we did observe some regional heterogeneity in the overall differences between daily minimum, maximum, and mean values. For example, in the Northeast, daily maximum and mean heat values have higher RRs for heat-associated hospitalizations than minimum values, whereas in the Southeast there is no notable difference between them. Although the drivers of this regional heterogeneity are outside the scope of this analysis, we postulate that they include differences in regional climate, individual time-activity patterns, cooling infrastructure, social determinants of health, and heat preparedness.

The lack of meaningful differences in RRs between the assessed variables nationwide was surprising, given that HI,^4^ WBGT,^7,8^ and UTCI^9^ were all specifically designed to have applicability in terms of human thermal comfort and/or physiological relevance. We considered a couple of hypotheses to explain their statistical equivalence with simple air temperature in identifying associations with heat-related health outcomes in these Medicare populations. First, we hypothesized that the heat metrics were strongly correlated at the high extreme, meaning that days at or above the 99th percentile were the same or similar regardless of the metric chosen. However, we found marked heterogeneity in the particular days that each metric identified as the “hottest” days over the period, suggesting that different “types” of hot days confer similar RRs of morbidity and mortality. Second, WBGT and UTCI are both designed as outdoor exposures, as they incorporate incident solar radiation and wind speed directly into their algorithms. For this reason, WBGT and UTCI have utility in active outdoor contexts, such as for athletes, military, and outdoor workers.^7,8^ However, the Medicare population is more likely to be socially isolated at home,^41,42^ so outdoor differences in WBGT or UTCI may be less applicable during both the daytime and nighttime.

Others have suggested that elevated overnight low temperatures are more dangerous to human health than daytime maximum temperatures,^43,44^ perhaps owing to warmer nighttime temperatures depriving the body of its ability to recover from the day’s heat stress. The data we presented in this article does not support this hypothesis: in almost all cases, across all regions, and for both all-cause mortality and heat-related hospitalizations, we found greater RRs associated with daytime maximum temperatures than with minimum temperatures, albeit with overlapping CI for the mortality measures. However, this does not mean that warm nighttime temperatures are not dangerous for health, nor does it preclude the possibility of an augmentative effect of elevated nighttime temperatures following days of extreme heat. In our modeling framework, we incorporated 5 days of lagged effects (i.e., heat on day 0 was associated with morbidity or mortality from days 0 through 5), meaning that the influence of overnight temperatures following the daytime extreme would be included and reflected in the pooled estimate. It is possible that this framework does not highlight an augmentative or interactive effect between daytime and nighttime temperatures. Moreover, comparisons between minimum and maximum temperatures reflect somewhat different timescales because the former generally occurs just before sunrise and the latter generally occurs in the early-to-mid afternoon.

Our study has additional limitations that may have affected the generalizability of our results. First, the climate data are at a spatial resolution of approximately 9 kilometers, which precludes the ability to resolve smaller-scale heterogeneity of exposures. WBGT and UTCI may have particularly important variations on these smaller scales, owing to things such as differences in the tree canopy and surface characteristics that influence ambient heat. Second, we assessed all heat-related hospitalizations together, though different relationships with the various heat metrics could theoretically exist for individual conditions. Finally, similar to all other nationwide analyses of heat, our study was unable to assess variability in individual-level exposures. This would importantly include things such as air conditioning utilization in the home or access to other air-conditioned spaces (e.g., cooling centers), which we were unable to control for in our models. It is possible that individual time-activity patterns would result in different relationships between the assessed heat metrics and health outcomes.

Despite these considerations, our findings provide compelling evidence that the choice of heat metric used to assess the health impacts of extreme heat in these particular US Medicare populations is not likely to substantively affect conclusions—it appears that hot days are similarly dangerous to health regardless of how they are defined and which “type” of heat metric is used to define them. This suggests that current interventions for older Americans during periods of extreme heat could be triggered by any measure of heat that is preferable. However, these findings are not necessarily applicable or transferable to the general population; additional research needs to be carried out to determine whether RRs of heat are similar between different heat metrics in younger Americans and susceptible subpopulations, including outdoor workers, children, and athletes, all of whom may be exposed to more direct sunshine and have the compounding factor of physical exertion that makes the heat more dangerous.

## Supplementary Material
