# Supplementary material for: Does choice of outdoor heat metric affect heat-related epidemiologic analyses in the US Medicare population?
Source: Environ Epidemiol. 2023 Jul 13;7(4):e261. doi: 10.1097/EE9.0000000000000261 (PMC10402938; doi:10.1097/EE9.0000000000000261)

Supplemental Digital Content

Does Choice of Outdoor Heat Metric Affect Heat-Related Epidemiologic Analyses in the US Medicare Population?

Keith R. Spangler[1*], Quinn H. Adams[1], Kate Hu[2], Danielle Braun[2,3], Kate R. Weinberger[4], Francesca Dominici[2], and Gregory A. Wellenius[1]

[1] Boston University School of Public Health, Dept. of Environmental Health, Boston, MA, USA

[2] Harvard University T.H. Chan School of Public Health, Dept. of Biostatistics, Boston, MA, USA

[3] Dana-Farber Cancer Institute, Dept of Data Science, Boston, MA, USA

[4] University of British Columbia, School of Population and Public Health, Vancouver, BC, Canada

[*] Corresponding author (krspangl@bu.edu)

Table of Contents

1. Table S1: Relative Risks of Mortality and Morbidity by Heat Metric and NCA Region..........................1
2. Figures S1-S7: Tables of Concordance between Heat Metrics by NCA Region.....................................4
3. Figures S8-S14: Forest Plots of Relative Risks by NCA Region at **99th** Percentile.................................8
4. Figures S15-S22: Forest Plots of Relative Risks by NCA Region at **95th** Percentile..............................15

Table S1 – Relative risks (RR) and 95% confidence intervals of all-cause mortality and heat-related hospitalizations at the 99th percentile of the heat metric indicated, among US Medicare enrollees (aged 65 years or older) nationally and by National Climate Assessment region from 2006-2016. RRs are relative to the heat metric percentile of minimum mortality/morbidity. **Bold** font indicates that the RR is statistically significant at the 5% level of p-values prior to rounding to two decimal places.

Region	Heat Metric	RR Mortality	RR Hospitalization
**National**	Max. Temperature	**1.03 (1.02, 1.04)**	**1.13 (1.12, 1.14)**
	Max. Heat Index	**1.03 (1.02, 1.04)**	**1.14 (1.12, 1.15)**
	Max. WBGT	**1.03 (1.02, 1.04)**	**1.12 (1.11, 1.14)**
	Max. UTCI	**1.03 (1.02, 1.04)**	**1.13 (1.11, 1.14)**
	Mean Temperature	**1.03 (1.02, 1.04)**	**1.14 (1.12, 1.15)**
	Mean Heat Index	**1.03 (1.02, 1.04)**	**1.14 (1.12, 1.15)**
	Mean WBGT	**1.03 (1.02, 1.04)**	**1.13 (1.12, 1.14)**
	Mean UTCI	**1.03 (1.02, 1.04)**	**1.14 (1.12, 1.15)**
	Min. Temperature	**1.02 (1.01, 1.03)**	**1.10 (1.09, 1.12)**
	Min. Heat Index	**1.02 (1.01, 1.03)**	**1.10 (1.09, 1.11)**
	Min. WBGT	**1.02 (1.01, 1.03)**	**1.09 (1.08, 1.11)**
	Min. UTCI	**1.02 (1.01, 1.03)**	**1.10 (1.08, 1.11)**
**Northeast**	Max. Temperature	**1.03 (1.01, 1.05)**	**1.18 (1.15, 1.21)**
	Max. Heat Index	**1.03 (1.01, 1.05)**	**1.19 (1.16, 1.21)**
	Max. WBGT	**1.04 (1.02, 1.06)**	**1.18 (1.15, 1.21)**
	Max. UTCI	**1.03 (1.01, 1.05)**	**1.18 (1.15, 1.21)**
	Mean Temperature	**1.03 (1.01, 1.05)**	**1.19 (1.16, 1.22)**
	Mean Heat Index	**1.03 (1.01, 1.05)**	**1.18 (1.15, 1.21)**
	Mean WBGT	**1.03 (1.01, 1.05)**	**1.18 (1.16, 1.21)**
	Mean UTCI	**1.03 (1.01, 1.05)**	**1.19 (1.16, 1.22)**
	Min. Temperature	1.01 (0.99, 1.03)	**1.14 (1.11, 1.17)**
	Min. Heat Index	1.01 (0.99, 1.03)	**1.13 (1.11, 1.16)**
	Min. WBGT	1.01 (0.99, 1.03)	**1.12 (1.10, 1.15)**
	Min. UTCI	1.01 (0.99, 1.03)	**1.12 (1.10, 1.14)**
**Southeast**	Max. Temperature	**1.02 (1.00, 1.04)**	**1.06 (1.04, 1.08)**
	Max. Heat Index	**1.03 (1.01, 1.05)**	**1.07 (1.05, 1.09)**
	Max. WBGT	1.01 (0.99, 1.03)	**1.05 (1.03, 1.08)**
	Max. UTCI	**1.02 (1.00, 1.04)**	**1.06 (1.04, 1.08)**
	Mean Temperature	**1.02 (1.00, 1.04)**	**1.08 (1.06, 1.11)**
	Mean Heat Index	**1.03 (1.01, 1.05)**	**1.08 (1.06, 1.10)**
	Mean WBGT	1.02 (1.00, 1.04)	**1.08 (1.06, 1.10)**
	Mean UTCI	**1.02 (1.00, 1.04)**	**1.08 (1.06, 1.10)**
	Min. Temperature	**1.02 (1.00, 1.04)**	**1.07 (1.04, 1.09)**
	Min. Heat Index	1.02 (1.00, 1.03)	**1.06 (1.04, 1.08)**
	Min. WBGT	1.02 (1.00, 1.04)	**1.06 (1.03, 1.08)**
	Min. UTCI	1.00 (0.98, 1.02)	**1.06 (1.03, 1.08)**

Region	Heat Metric	RR Mortality	RR Hospitalization
**Midwest**	Max. Temperature	**1.03 (1.01, 1.05)**	**1.13 (1.10, 1.16)**
	Max. Heat Index	**1.02 (1.00, 1.05)**	**1.13 (1.10, 1.16)**
	Max. WBGT	1.02 (1.00, 1.04)	**1.12 (1.09, 1.15)**
	Max. UTCI	**1.03 (1.00, 1.05)**	**1.12 (1.09, 1.15)**
	Mean Temperature	**1.03 (1.01, 1.05)**	**1.13 (1.10, 1.16)**
	Mean Heat Index	**1.02 (1.00, 1.04)**	**1.12 (1.09, 1.16)**
	Mean WBGT	**1.02 (1.00, 1.04)**	**1.12 (1.09, 1.15)**
	Mean UTCI	**1.02 (1.00, 1.04)**	**1.13 (1.10, 1.16)**
	Min. Temperature	1.02 (1.00, 1.05)	**1.09 (1.06, 1.12)**
	Min. Heat Index	1.02 (1.00, 1.04)	**1.09 (1.06, 1.12)**
	Min. WBGT	**1.02 (1.00, 1.05)**	**1.09 (1.06, 1.12)**
	Min. UTCI	1.02 (1.00, 1.04)	**1.09 (1.07, 1.12)**
**Northern Great Plains**	Max. Temperature	0.98 (0.88, 1.09)	1.03 (0.87, 1.21)
	Max. Heat Index	0.96 (0.86, 1.07)	1.04 (0.90, 1.21)
	Max. WBGT	0.97 (0.85, 1.11)	0.99 (0.84, 1.18)
	Max. UTCI	0.98 (0.89, 1.08)	1.02 (0.89, 1.17)
	Mean Temperature	0.98 (0.88, 1.08)	1.00 (0.87, 1.16)
	Mean Heat Index	0.96 (0.88, 1.06)	1.03 (0.90, 1.18)
	Mean WBGT	0.96 (0.88, 1.06)	0.95 (0.83, 1.09)
	Mean UTCI	0.97 (0.87, 1.07)	0.97 (0.81, 1.18)
	Min. Temperature	1.01 (0.89, 1.13)	0.88 (0.74, 1.04)
	Min. Heat Index	0.99 (0.89, 1.11)	0.89 (0.75, 1.05)
	Min. WBGT	0.94 (0.85, 1.04)	0.89 (0.75, 1.05)
	Min. UTCI	0.95 (0.86, 1.05)	0.91 (0.77, 1.06)
**Southern Great Plains**	Max. Temperature	**1.05 (1.01, 1.08)**	**1.09 (1.05, 1.13)**
	Max. Heat Index	1.03 (0.99, 1.06)	**1.11 (1.07, 1.14)**
	Max. WBGT	1.01 (0.97, 1.06)	**1.07 (1.03, 1.12)**
	Max. UTCI	**1.04 (1.01, 1.08)**	**1.08 (1.04, 1.12)**
	Mean Temperature	**1.06 (1.02, 1.09)**	**1.10 (1.06, 1.14)**
	Mean Heat Index	1.03 (0.99, 1.06)	**1.10 (1.06, 1.14)**
	Mean WBGT	1.01 (0.97, 1.04)	**1.09 (1.05, 1.13)**
	Mean UTCI	**1.04 (1.01, 1.08)**	**1.10 (1.06, 1.14)**
	Min. Temperature	**1.04 (1.01, 1.08)**	**1.06 (1.02, 1.11)**
	Min. Heat Index	1.02 (0.98, 1.05)	**1.06 (1.02, 1.11)**
	Min. WBGT	0.99 (0.96, 1.02)	**1.05 (1.01, 1.08)**
	Min. UTCI	1.01 (0.98, 1.05)	**1.06 (1.02, 1.10)**

Region	Heat Metric	RR Mortality	RR Hospitalization
**Southwest**	Max. Temperature	**1.06 (1.04, 1.08)**	**1.15 (1.12, 1.19)**
	Max. Heat Index	**1.06 (1.04, 1.08)**	**1.17 (1.13, 1.21)**
	Max. WBGT	**1.05 (1.03, 1.08)**	**1.16 (1.11, 1.20)**
	Max. UTCI	**1.06 (1.04, 1.09)**	**1.16 (1.12, 1.20)**
	Mean Temperature	**1.05 (1.03, 1.08)**	**1.16 (1.12, 1.20)**
	Mean Heat Index	**1.05 (1.03, 1.07)**	**1.16 (1.12, 1.20)**
	Mean WBGT	**1.04 (1.02, 1.06)**	**1.13 (1.09, 1.17)**
	Mean UTCI	**1.05 (1.03, 1.07)**	**1.16 (1.11, 1.20)**
	Min. Temperature	**1.05 (1.03, 1.07)**	**1.12 (1.08, 1.16)**
	Min. Heat Index	**1.04 (1.02, 1.06)**	**1.11 (1.08, 1.15)**
	Min. WBGT	**1.03 (1.01, 1.05)**	**1.10 (1.06, 1.13)**
	Min. UTCI	**1.04 (1.02, 1.06)**	**1.10 (1.05, 1.15)**
**Northwest**	Max. Temperature	**1.05 (1.00, 1.10)**	**1.17 (1.08, 1.27)**
	Max. Heat Index	**1.05 (1.01, 1.10)**	**1.19 (1.09, 1.29)**
	Max. WBGT	**1.08 (1.03, 1.13)**	**1.20 (1.09, 1.31)**
	Max. UTCI	**1.05 (1.01, 1.10)**	**1.17 (1.08, 1.27)**
	Mean Temperature	1.04 (1.00, 1.09)	**1.17 (1.08, 1.27)**
	Mean Heat Index	**1.04 (1.00, 1.09)**	**1.18 (1.09, 1.27)**
	Mean WBGT	**1.06 (1.01, 1.10)**	**1.19 (1.10, 1.28)**
	Mean UTCI	**1.05 (1.00, 1.09)**	**1.17 (1.09, 1.26)**
	Min. Temperature	1.03 (0.99, 1.08)	**1.14 (1.06, 1.23)**
	Min. Heat Index	1.03 (0.99, 1.08)	**1.15 (1.06, 1.24)**
	Min. WBGT	1.04 (1.00, 1.09)	**1.16 (1.08, 1.25)**
	Min. UTCI	1.04 (0.99, 1.09)	**1.15 (1.07, 1.24)**

Figures S1-S7. Concordance between extreme-heat days by metric by NCA region. Values indicate the percentage of days in which the top 1% hottest days by the given metric (columns) were in the top 2% of hottest days by the other metric (rows) for the counties included in the analysis over the warm seasons (May-September) of 2006-2016. Abbreviations and acronyms: min. = minimum, max. = maximum, temp. = air temperature, WBGT = wet-bulb globe temperature, UTCI = Universal Thermal Climate Index, HI = heat index.

Figure S1

Northeast		Maximum				Mean				Minimum			
		Temp	HI	WBGT	UTCI	Temp	HI	WBGT	UTCI	Temp	HI	WBGT	UTCI
Maximum	Temp	100%	69%	37%	84%	88%	69%	50%	65%	42%	41%	31%	31%
	HI	69%	100%	54%	72%	74%	90%	80%	79%	63%	65%	59%	55%
	WBGT	37%	54%	100%	61%	40%	50%	64%	58%	33%	34%	35%	37%
	UTCI	84%	72%	61%	100%	76%	70%	63%	77%	41%	41%	34%	36%
Mean	Temp	88%	74%	40%	76%	100%	91%	68%	86%	65%	63%	47%	47%
	HI	69%	90%	50%	70%	91%	100%	91%	95%	78%	79%	67%	64%
	WBGT	50%	80%	64%	63%	68%	91%	100%	94%	67%	70%	72%	70%
	UTCI	65%	79%	58%	77%	86%	95%	94%	100%	67%	68%	61%	65%
Minimum	Temp	42%	63%	33%	41%	65%	78%	67%	67%	100%	100%	79%	73%
	HI	41%	65%	34%	41%	63%	79%	70%	68%	100%	100%	87%	79%
	WBGT	31%	59%	35%	34%	47%	67%	72%	61%	79%	87%	100%	89%
	UTCI	31%	55%	37%	36%	47%	64%	70%	65%	73%	79%	89%	100%

Figure S2

Midwest		Maximum				Mean				Minimum			
		Temp	HI	WBGT	UTCI	Temp	HI	WBGT	UTCI	Temp	HI	WBGT	UTCI
Maximum	Temp	100%	64%	34%	79%	90%	66%	44%	59%	55%	51%	33%	33%
	HI	64%	100%	50%	64%	64%	85%	73%	65%	57%	59%	56%	50%
	WBGT	34%	50%	100%	61%	34%	46%	63%	58%	31%	33%	37%	42%
	UTCI	79%	64%	61%	100%	71%	66%	59%	76%	47%	46%	37%	42%
Mean	Temp	90%	64%	34%	71%	100%	84%	57%	71%	78%	73%	45%	44%
	HI	66%	85%	46%	66%	84%	100%	86%	85%	80%	83%	68%	63%
	WBGT	44%	73%	63%	59%	57%	86%	100%	88%	59%	66%	77%	74%
	UTCI	59%	65%	58%	76%	71%	85%	88%	100%	60%	64%	61%	71%
Minimum	Temp	55%	57%	31%	47%	78%	80%	59%	60%	100%	99%	65%	57%
	HI	51%	59%	33%	46%	73%	83%	66%	64%	99%	100%	77%	66%
	WBGT	33%	56%	37%	37%	45%	68%	77%	61%	65%	77%	100%	85%
	UTCI	33%	50%	42%	42%	44%	63%	74%	71%	57%	66%	85%	100%

Figure S3

Southeast		Maximum				Mean				Minimum			
		Temp	HI	WBGT	UTCI	Temp	HI	WBGT	UTCI	Temp	HI	WBGT	UTCI
Maximum	Temp	100%	39%	18%	76%	81%	43%	23%	44%	33%	29%	14%	15%
	HI	39%	100%	34%	53%	46%	78%	66%	67%	47%	50%	48%	46%
	WBGT	18%	34%	100%	37%	19%	29%	48%	36%	17%	19%	21%	24%
	UTCI	76%	53%	37%	100%	69%	53%	40%	60%	35%	33%	22%	24%
Mean	Temp	81%	46%	19%	69%	100%	65%	35%	62%	50%	45%	24%	24%
	HI	43%	78%	29%	53%	65%	100%	75%	86%	66%	69%	58%	53%
	WBGT	23%	66%	48%	40%	35%	75%	100%	81%	40%	46%	56%	58%
	UTCI	44%	67%	36%	60%	62%	86%	81%	100%	48%	50%	46%	54%
Minimum	Temp	33%	47%	17%	35%	50%	66%	40%	48%	100%	98%	56%	44%
	HI	29%	50%	19%	33%	45%	69%	46%	50%	98%	100%	69%	54%
	WBGT	14%	48%	21%	22%	24%	58%	56%	46%	56%	69%	100%	76%
	UTCI	15%	46%	24%	24%	24%	53%	58%	54%	44%	54%	76%	100%

Figure S4

NGP		Maximum				Mean				Minimum			
		Temp	HI	WBGT	UTCI	Temp	HI	WBGT	UTCI	Temp	HI	WBGT	UTCI
Maximum	Temp	100%	60%	19%	80%	86%	69%	28%	61%	42%	40%	16%	21%
	HI	60%	100%	37%	64%	60%	77%	58%	58%	34%	36%	30%	22%
	WBGT	19%	37%	100%	39%	22%	32%	52%	38%	19%	21%	20%	23%
	UTCI	80%	64%	39%	100%	71%	69%	44%	76%	41%	39%	18%	25%
Mean	Temp	86%	60%	22%	71%	100%	86%	37%	81%	70%	67%	24%	34%
	HI	69%	77%	32%	69%	86%	100%	59%	81%	66%	69%	39%	40%
	WBGT	28%	58%	52%	44%	37%	59%	100%	63%	31%	38%	56%	44%
	UTCI	61%	58%	38%	76%	81%	81%	63%	100%	55%	54%	34%	50%
Minimum	Temp	42%	34%	19%	41%	70%	66%	31%	55%	100%	99%	34%	46%
	HI	40%	36%	21%	39%	67%	69%	38%	54%	99%	100%	48%	53%
	WBGT	16%	30%	20%	18%	24%	39%	56%	34%	34%	48%	100%	59%
	UTCI	21%	22%	23%	25%	34%	40%	44%	50%	46%	53%	59%	100%

Figure S5

SGP		Maximum				Mean				Minimum			
		Temp	HI	WBGT	UTCI	Temp	HI	WBGT	UTCI	Temp	HI	WBGT	UTCI
Maximum	Temp	100%	45%	13%	85%	79%	41%	14%	53%	40%	28%	10%	15%
	HI	45%	100%	20%	49%	45%	74%	43%	47%	38%	42%	28%	27%
	WBGT	13%	20%	100%	23%	13%	20%	39%	25%	12%	13%	12%	18%
	UTCI	85%	49%	23%	100%	71%	49%	23%	63%	38%	30%	12%	17%
Mean	Temp	79%	45%	13%	71%	100%	56%	19%	70%	64%	44%	12%	22%
	HI	41%	74%	20%	49%	56%	100%	53%	66%	59%	73%	43%	43%
	WBGT	14%	43%	39%	23%	19%	53%	100%	40%	21%	36%	50%	42%
	UTCI	53%	47%	25%	63%	70%	66%	40%	100%	52%	46%	21%	46%
Minimum	Temp	40%	38%	12%	38%	64%	59%	21%	52%	100%	75%	25%	33%
	HI	28%	42%	13%	30%	44%	73%	36%	46%	75%	100%	51%	49%
	WBGT	10%	28%	12%	12%	12%	43%	50%	21%	25%	51%	100%	44%
	UTCI	15%	27%	18%	17%	22%	43%	42%	46%	33%	49%	44%	100%

Figure S6

Southwest		Maximum				Mean				Minimum			
		Temp	HI	WBGT	UTCI	Temp	HI	WBGT	UTCI	Temp	HI	WBGT	UTCI
Maximum	Temp	100%	91%	26%	88%	66%	49%	14%	45%	12%	9%	2%	3%
	HI	91%	100%	33%	91%	67%	65%	21%	54%	17%	15%	5%	7%
	WBGT	26%	33%	100%	35%	22%	25%	34%	26%	8%	9%	11%	7%
	UTCI	88%	91%	35%	100%	62%	53%	18%	51%	13%	12%	3%	4%
Mean	Temp	66%	67%	22%	62%	100%	93%	18%	69%	35%	30%	5%	12%
	HI	49%	65%	25%	53%	93%	100%	32%	84%	41%	40%	13%	21%
	WBGT	14%	21%	34%	18%	18%	32%	100%	42%	15%	23%	54%	34%
	UTCI	45%	54%	26%	51%	69%	84%	42%	100%	32%	33%	18%	22%
Minimum	Temp	12%	17%	8%	13%	35%	41%	15%	32%	100%	96%	14%	39%
	HI	9%	15%	9%	12%	30%	40%	23%	33%	96%	100%	27%	53%
	WBGT	2%	5%	11%	3%	5%	13%	54%	18%	14%	27%	100%	61%
	UTCI	3%	7%	7%	4%	12%	21%	34%	22%	39%	53%	61%	100%

Figure S7

Northwest		Maximum				Mean				Minimum			
		Temp	HI	WBGT	UTCI	Temp	HI	WBGT	UTCI	Temp	HI	WBGT	UTCI
Maximum	Temp	100%	97%	54%	97%	88%	81%	55%	76%	44%	42%	33%	33%
	HI	97%	100%	67%	98%	89%	89%	73%	88%	47%	46%	41%	40%
	WBGT	54%	67%	100%	65%	54%	59%	73%	63%	38%	39%	39%	36%
	UTCI	97%	98%	65%	100%	85%	84%	65%	83%	44%	42%	36%	35%
Mean	Temp	88%	89%	54%	85%	100%	100%	69%	95%	65%	62%	48%	49%
	HI	81%	89%	59%	84%	100%	100%	80%	99%	70%	68%	57%	56%
	WBGT	55%	73%	73%	65%	69%	80%	100%	87%	58%	61%	70%	60%
	UTCI	76%	88%	63%	83%	95%	99%	87%	100%	65%	65%	58%	57%
Minimum	Temp	44%	47%	38%	44%	65%	70%	58%	65%	100%	100%	73%	76%
	HI	42%	46%	39%	42%	62%	68%	61%	65%	100%	100%	80%	84%
	WBGT	33%	41%	39%	36%	48%	57%	70%	58%	73%	80%	100%	89%
	UTCI	33%	40%	36%	35%	49%	56%	60%	57%	76%	84%	89%	100%

Figure S8. Northeast NCA region relative risks (RR) of mortality (blue lines with squares) and heat-associated hospitalizations (green lines with circles) at the **99th percentile** of different heat metrics in the US Medicare population, 2006-2016. Abbreviations: "Max." = maximum and "Min." = minimum. Relative risks are for the 99th percentile of each heat metric compared to the corresponding heat metric of minimum morbidity/mortality.

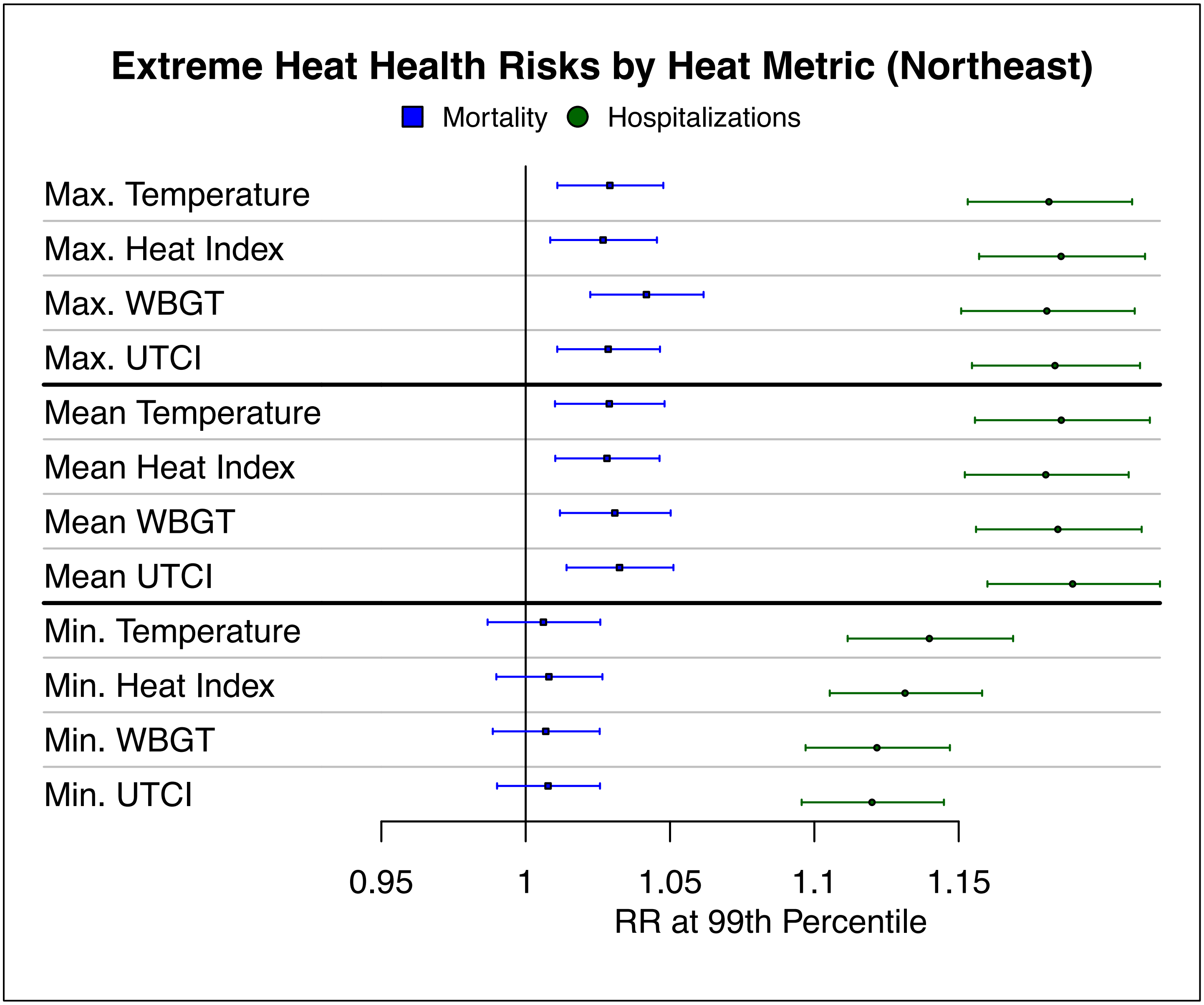

Figure S9. Southeast NCA region relative risks (RR) of mortality (blue lines with squares) and heat-associated hospitalizations (green lines with circles) at the **99th percentile** of different heat metrics in the US Medicare population, 2006-2016. Abbreviations: "Max." = maximum and "Min." = minimum. Relative risks are for the 99th percentile of each heat metric compared to the corresponding heat metric of minimum morbidity/mortality.

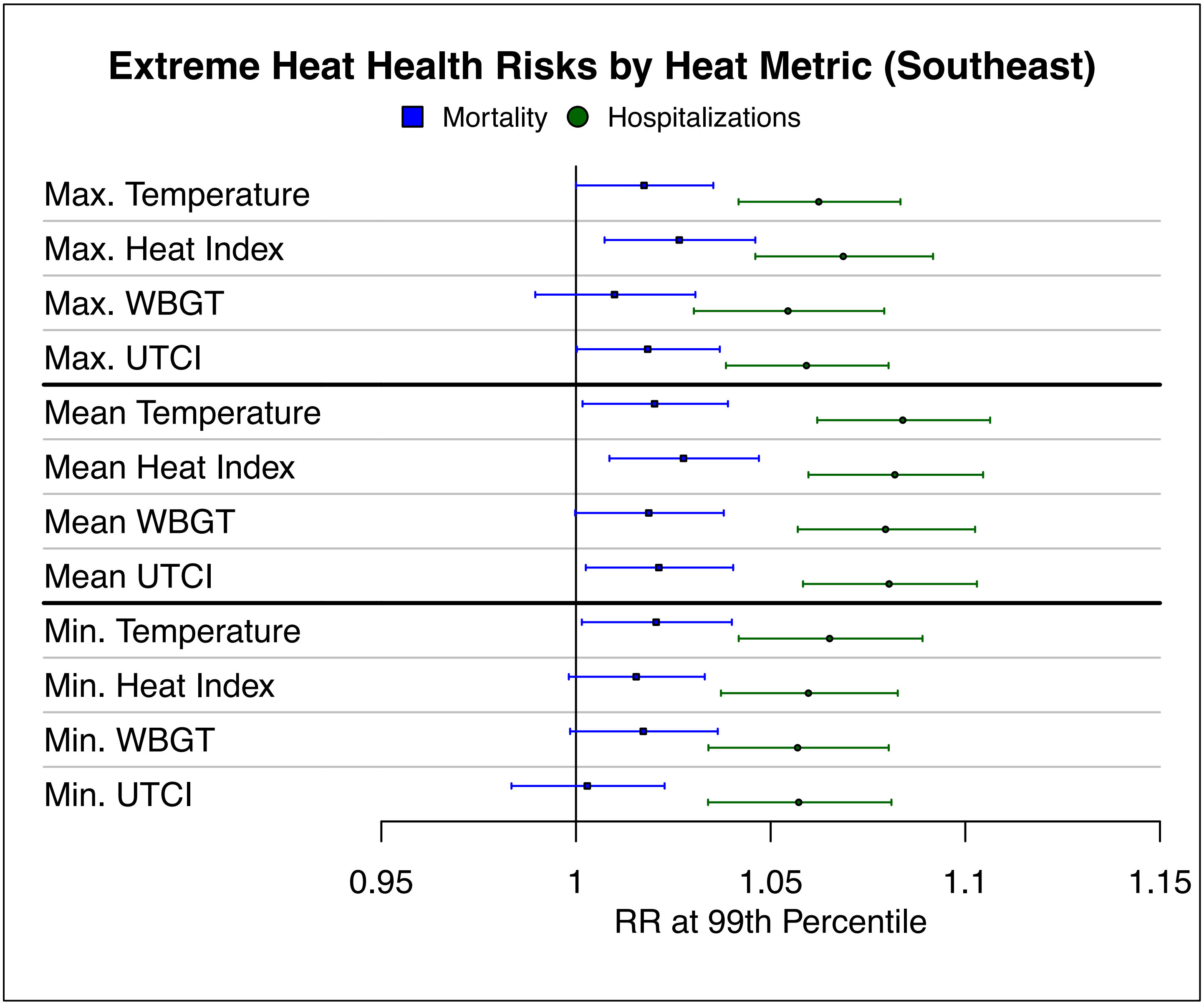

Figure S10. Midwest NCA region relative risks (RR) of mortality (blue lines with squares) and heat-associated hospitalizations (green lines with circles) at the **99th percentile** of different heat metrics in the US Medicare population, 2006-2016. Abbreviations: "Max." = maximum and "Min." = minimum. Relative risks are for the 99th percentile of each heat metric compared to the corresponding heat metric of minimum morbidity/mortality.

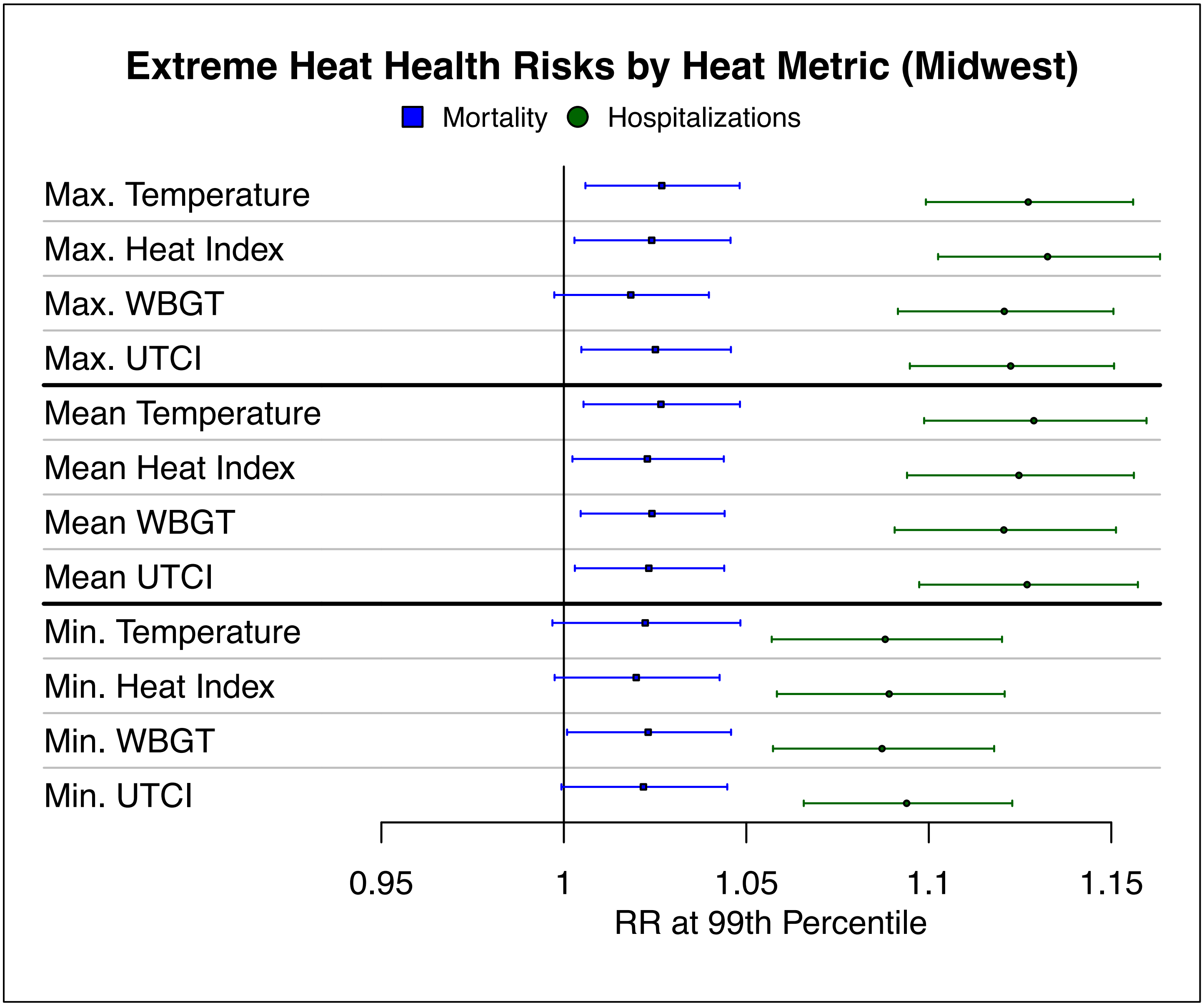

Figure S11. Southern Great Plains (SGP) NCA region relative risks (RR) of mortality (blue lines with squares) and heat-associated hospitalizations (green lines with circles) at the **99th percentile** of different heat metrics in the US Medicare population, 2006-2016. Abbreviations: "Max." = maximum and "Min." = minimum. Relative risks are for the 99th percentile of each heat metric compared to the corresponding heat metric of minimum morbidity/mortality.

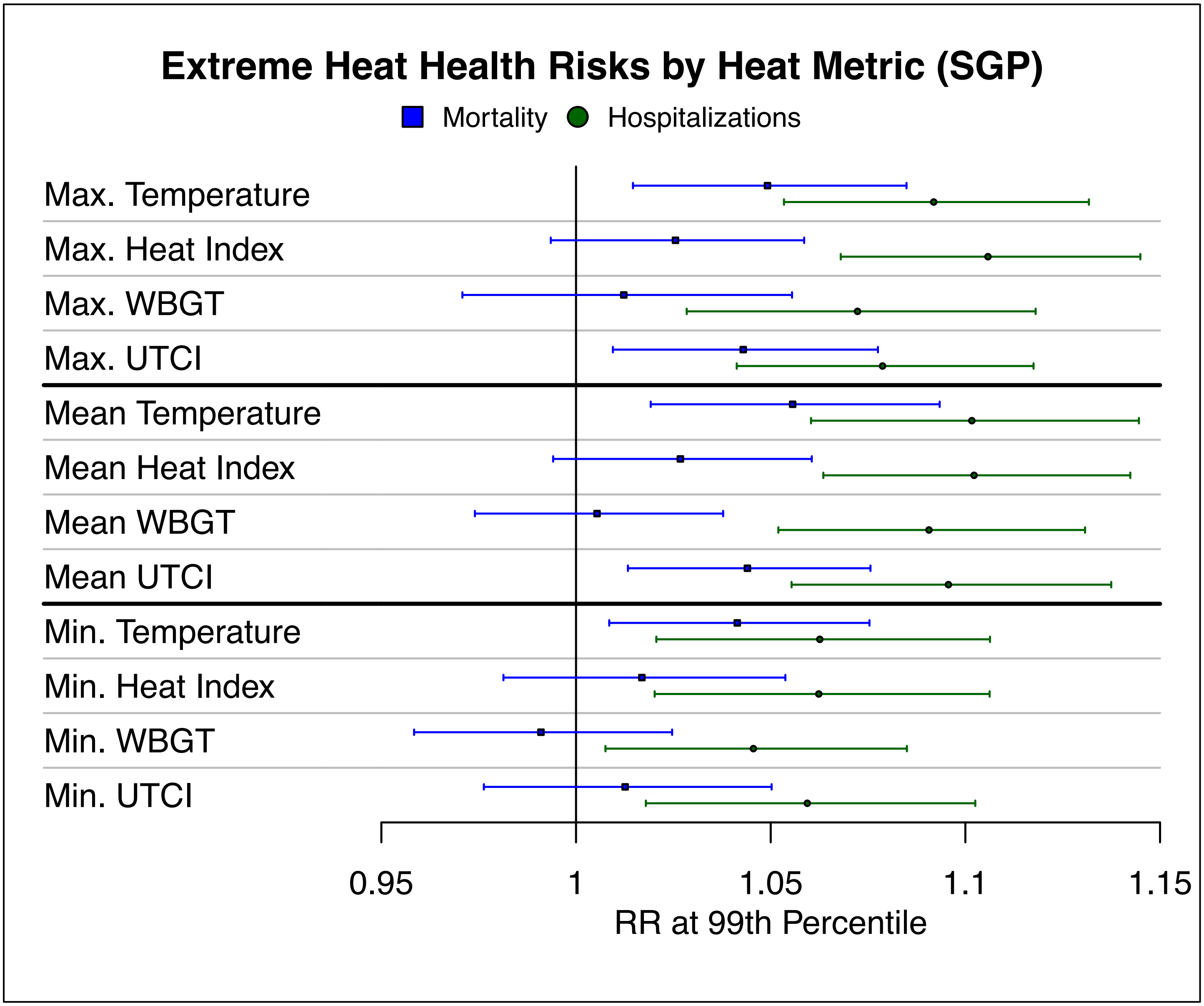

Figure S12. Northern Great Plains (NGP) NCA region relative risks (RR) of mortality (blue lines with squares) and heat-associated hospitalizations (green lines with circles) at the **99th percentile** of different heat metrics in the US Medicare population, 2006-2016. Abbreviations: "Max." = maximum and "Min." = minimum. Relative risks are for the 99th percentile of each heat metric compared to the corresponding heat metric of minimum morbidity/mortality.

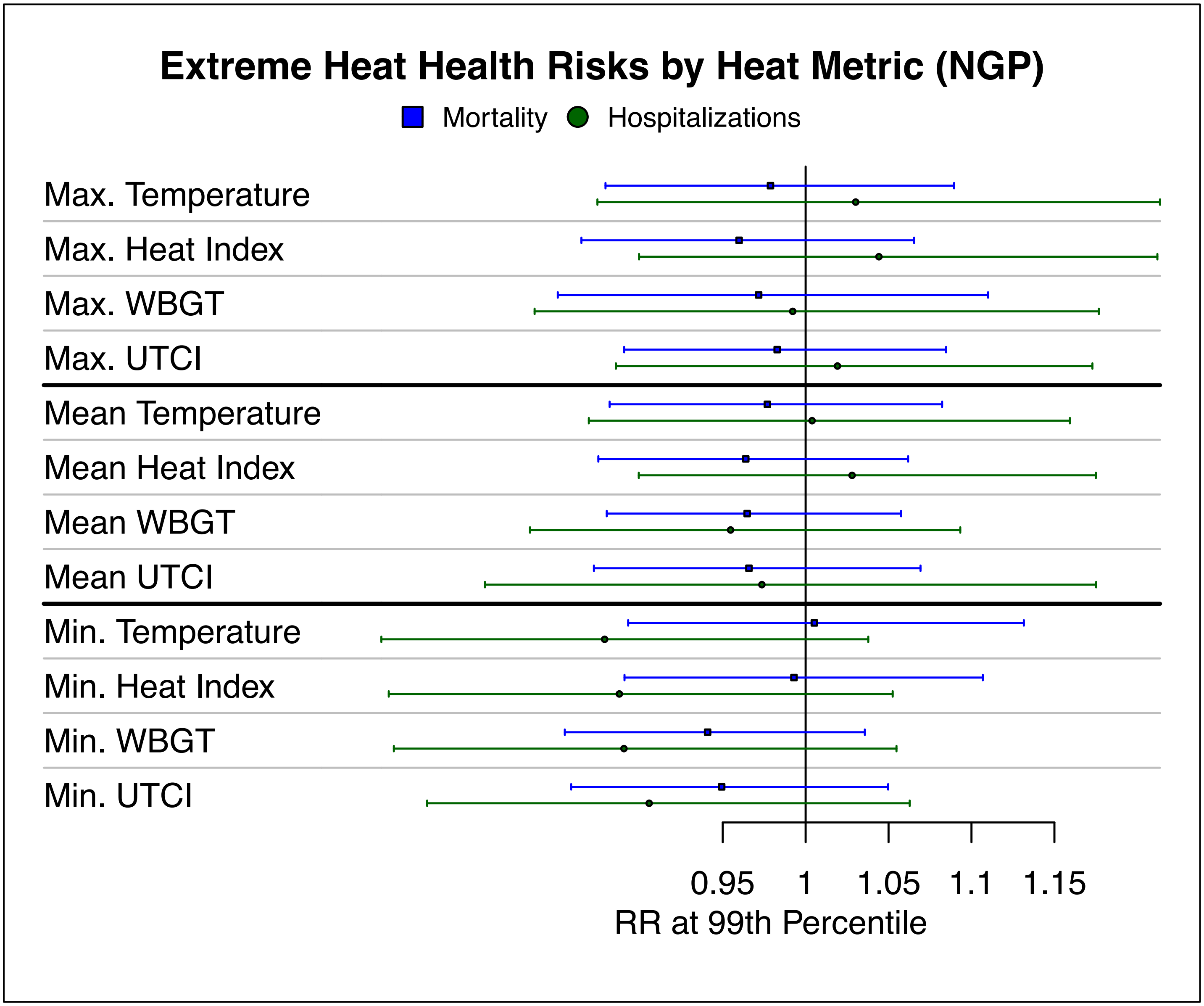

Figure S13. Southwest NCA region relative risks (RR) of mortality (blue lines with squares) and heat-associated hospitalizations (green lines with circles) at the **99th percentile** of different heat metrics in the US Medicare population, 2006-2016. Abbreviations: "Max." = maximum and "Min." = minimum. Relative risks are for the 99th percentile of each heat metric compared to the corresponding heat metric of minimum morbidity/mortality.

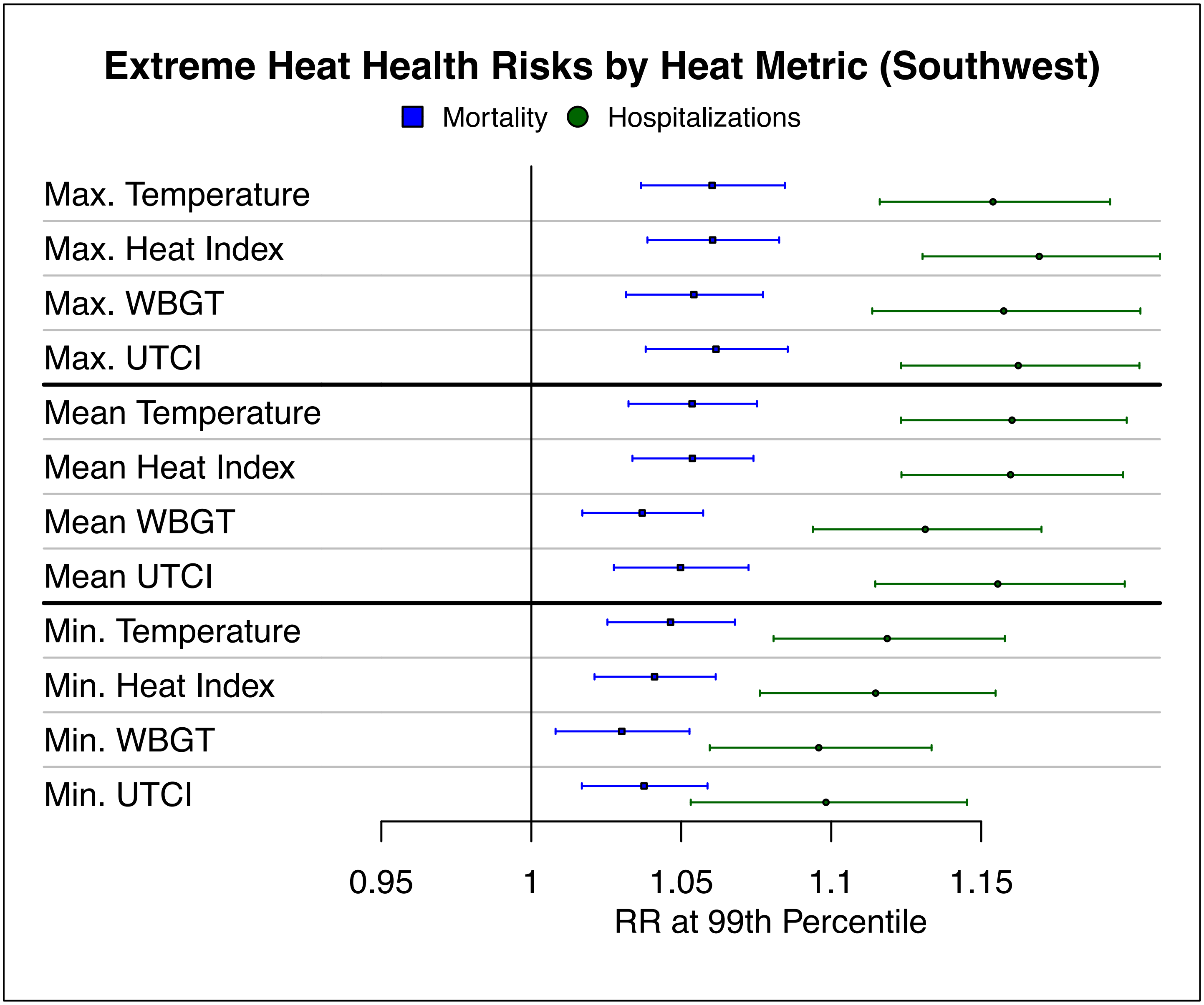

Figure S14. Northwest NCA region relative risks (RR) of mortality (blue lines with squares) and heat-associated hospitalizations (green lines with circles) at the **99th percentile** of different heat metrics in the US Medicare population, 2006-2016. Abbreviations: “Max.” = maximum and “Min.” = minimum. Relative risks are for the 99th percentile of each heat metric compared to the corresponding heat metric of minimum morbidity/mortality.

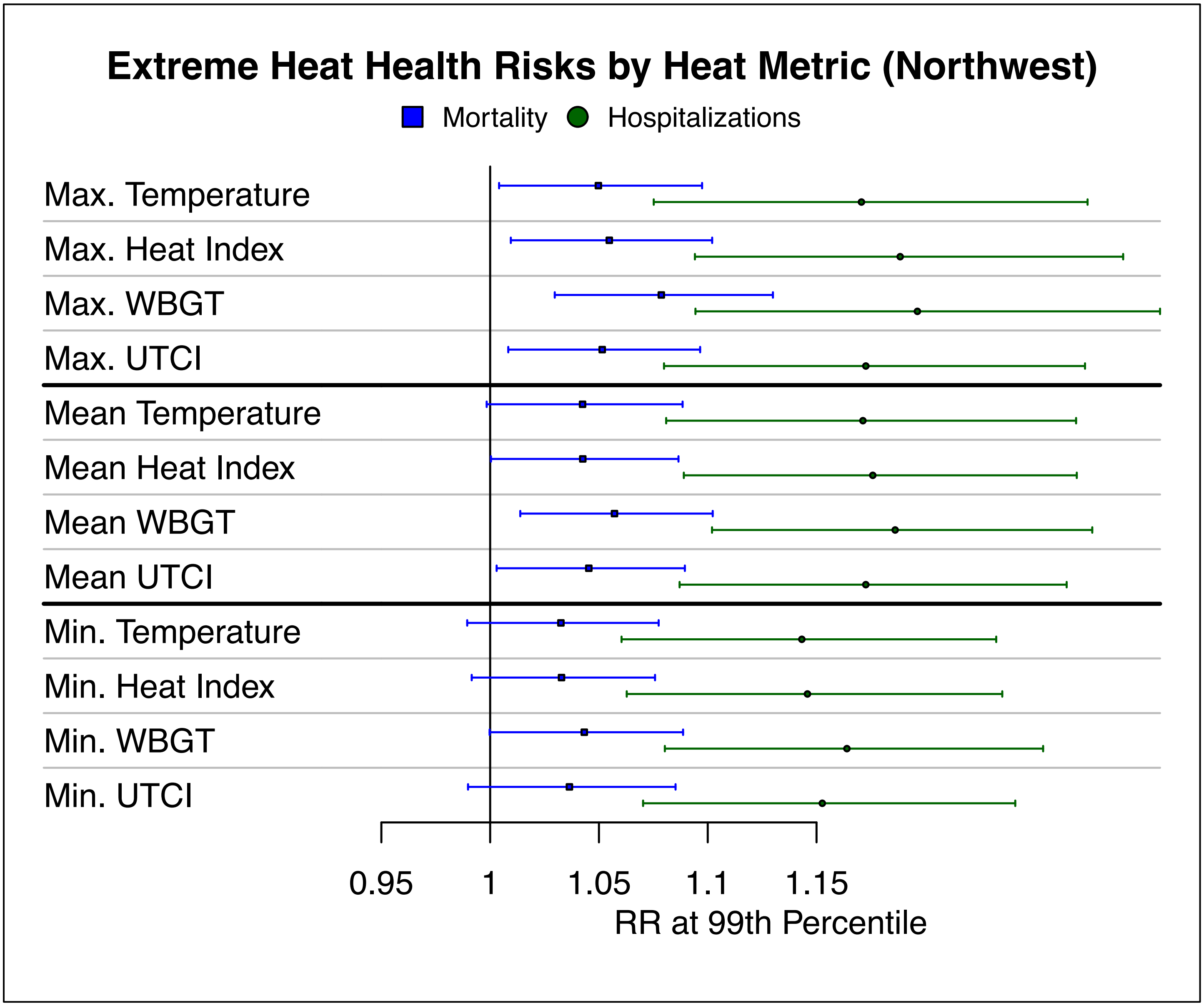

Figure S15. National relative risks (RR) of mortality (blue lines with squares) and heat-associated hospitalizations (green lines with circles) at the **95th percentile** of different heat metrics in the US Medicare population, 2006-2016. Abbreviations: "Max." = maximum and "Min." = minimum. Relative risks are for the 99th percentile of each heat metric compared to the corresponding heat metric of minimum morbidity/mortality.

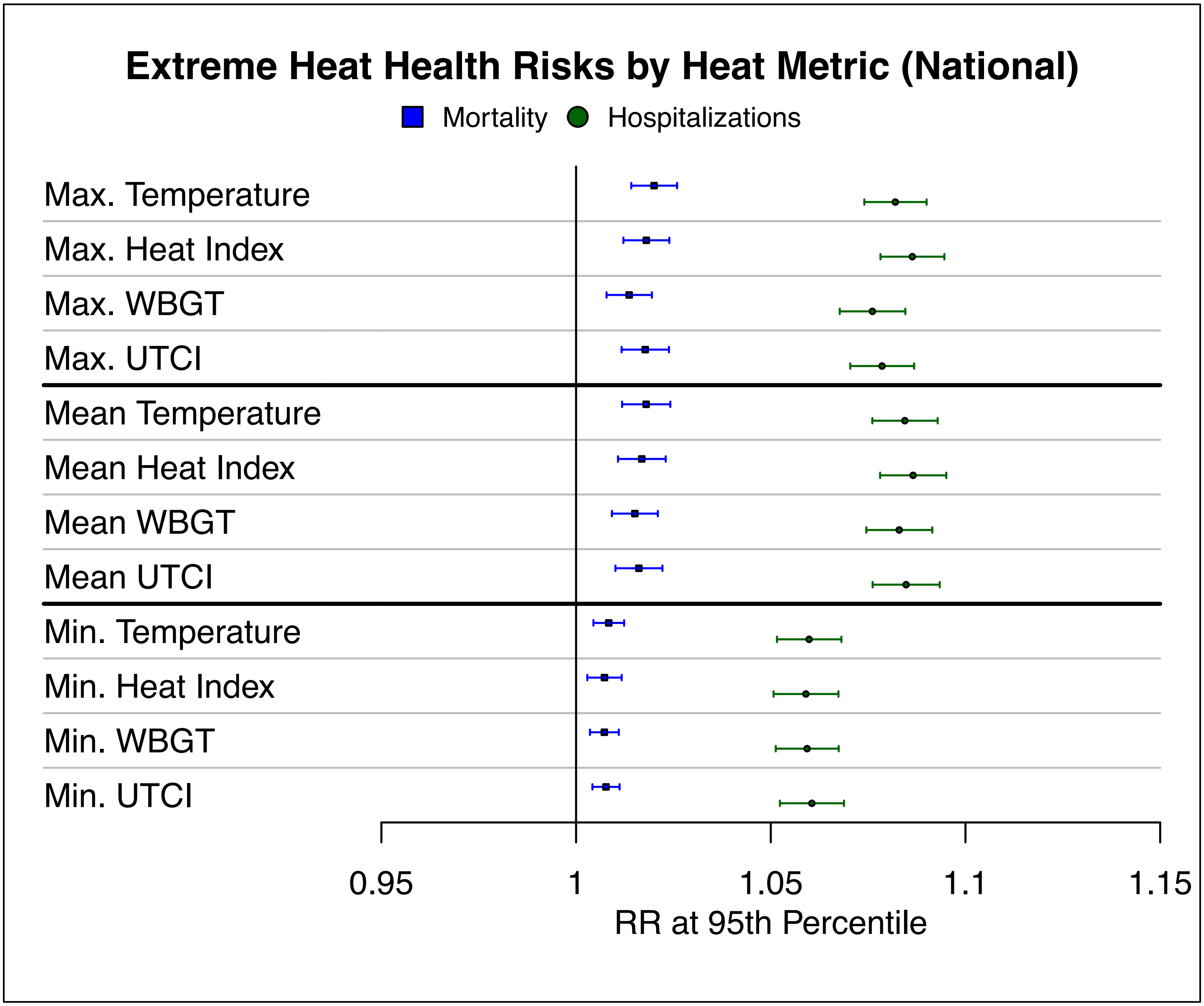

Figure S16. Northeast NCA region relative risks (RR) of mortality (blue lines with squares) and heat-associated hospitalizations (green lines with circles) at the **95th percentile** of different heat metrics in the US Medicare population, 2006-2016. Abbreviations: "Max." = maximum and "Min." = minimum. Relative risks are for the 99th percentile of each heat metric compared to the corresponding heat metric of minimum morbidity/mortality.

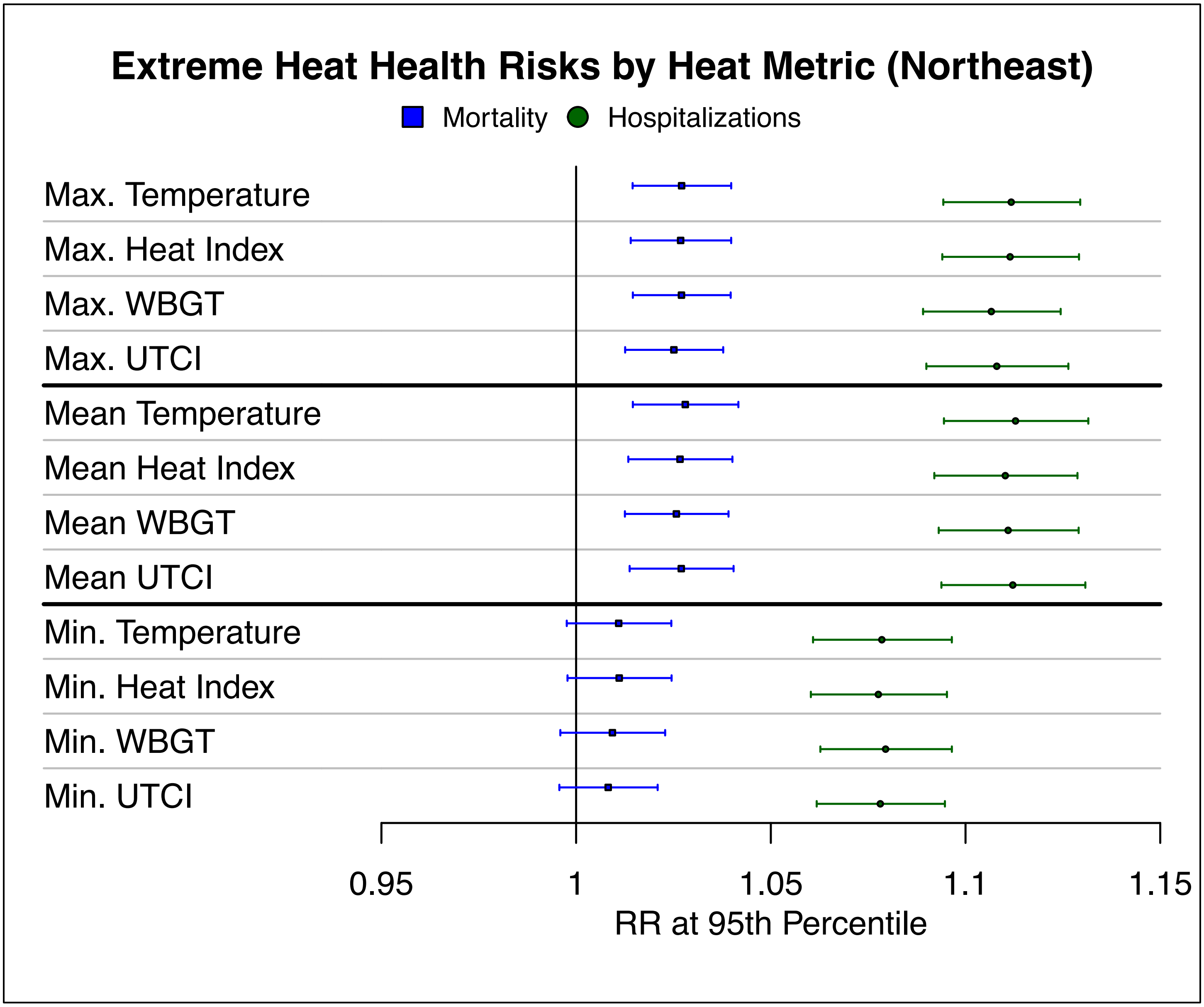

Figure S17. Southeast NCA region relative risks (RR) of mortality (blue lines with squares) and heat-associated hospitalizations (green lines with circles) at the **95th percentile** of different heat metrics in the US Medicare population, 2006-2016. Abbreviations: "Max." = maximum and "Min." = minimum. Relative risks are for the 99th percentile of each heat metric compared to the corresponding heat metric of minimum morbidity/mortality.

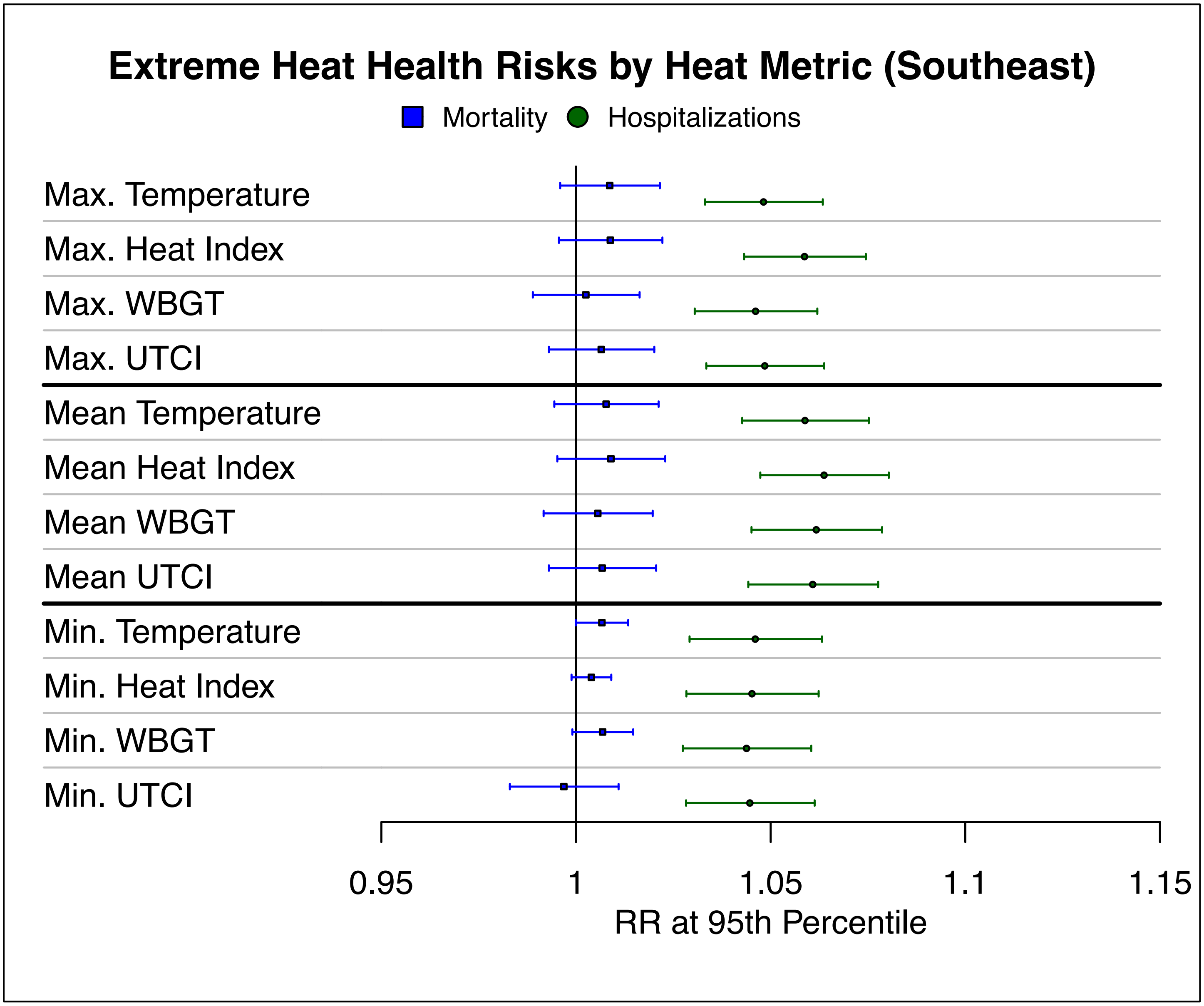

Figure S18. Midwest NCA region relative risks (RR) of mortality (blue lines with squares) and heat-associated hospitalizations (green lines with circles) at the **95th percentile** of different heat metrics in the US Medicare population, 2006-2016. Abbreviations: "Max." = maximum and "Min." = minimum. Relative risks are for the 99th percentile of each heat metric compared to the corresponding heat metric of minimum morbidity/mortality.

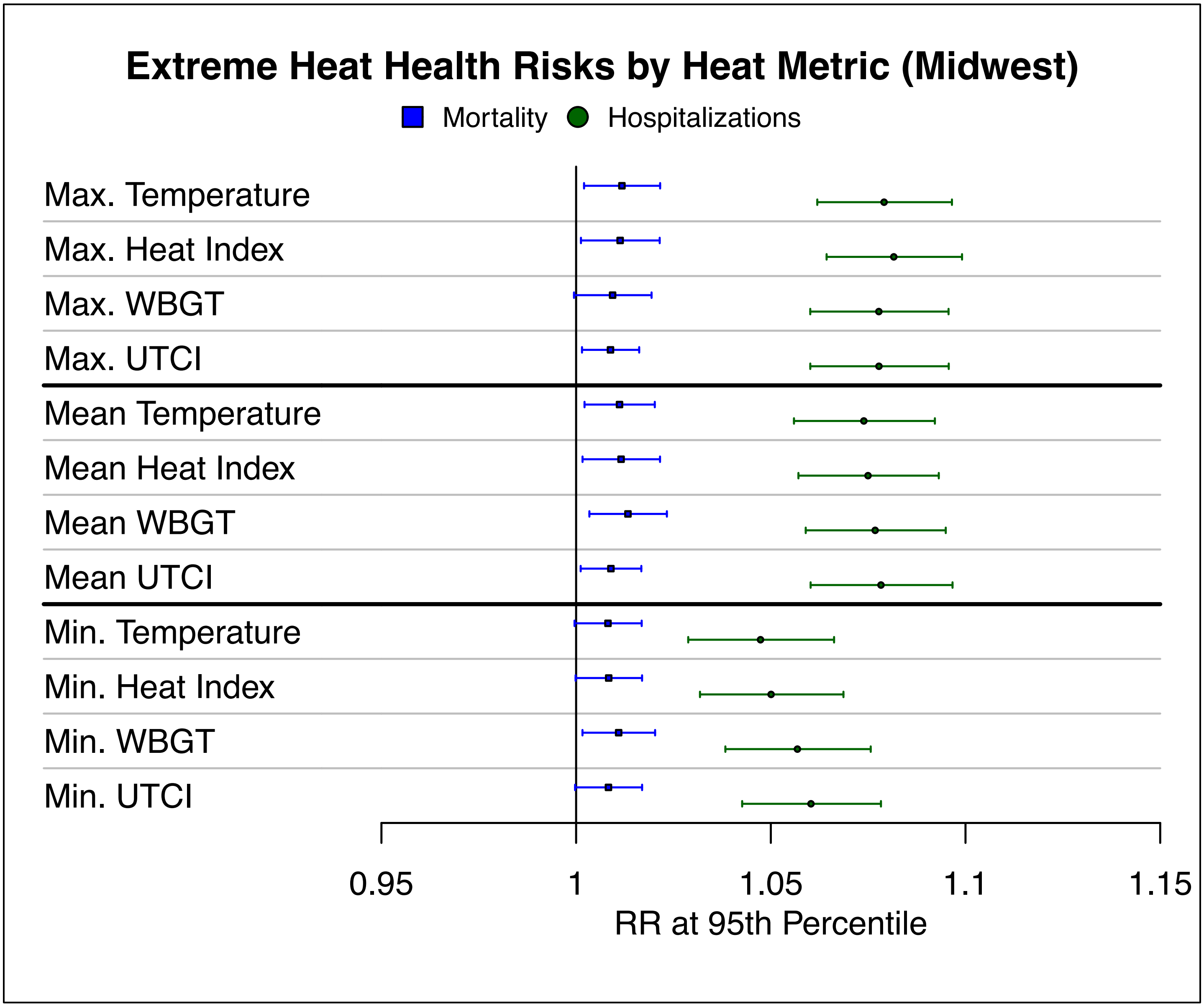

Figure S19. Northern Great Plains NCA region relative risks (RR) of mortality (blue lines with squares) and heat-associated hospitalizations (green lines with circles) at the **95th percentile** of different heat metrics in the US Medicare population, 2006-2016. Abbreviations: "Max." = maximum and "Min." = minimum. Relative risks are for the 99th percentile of each heat metric compared to the corresponding heat metric of minimum morbidity/mortality.

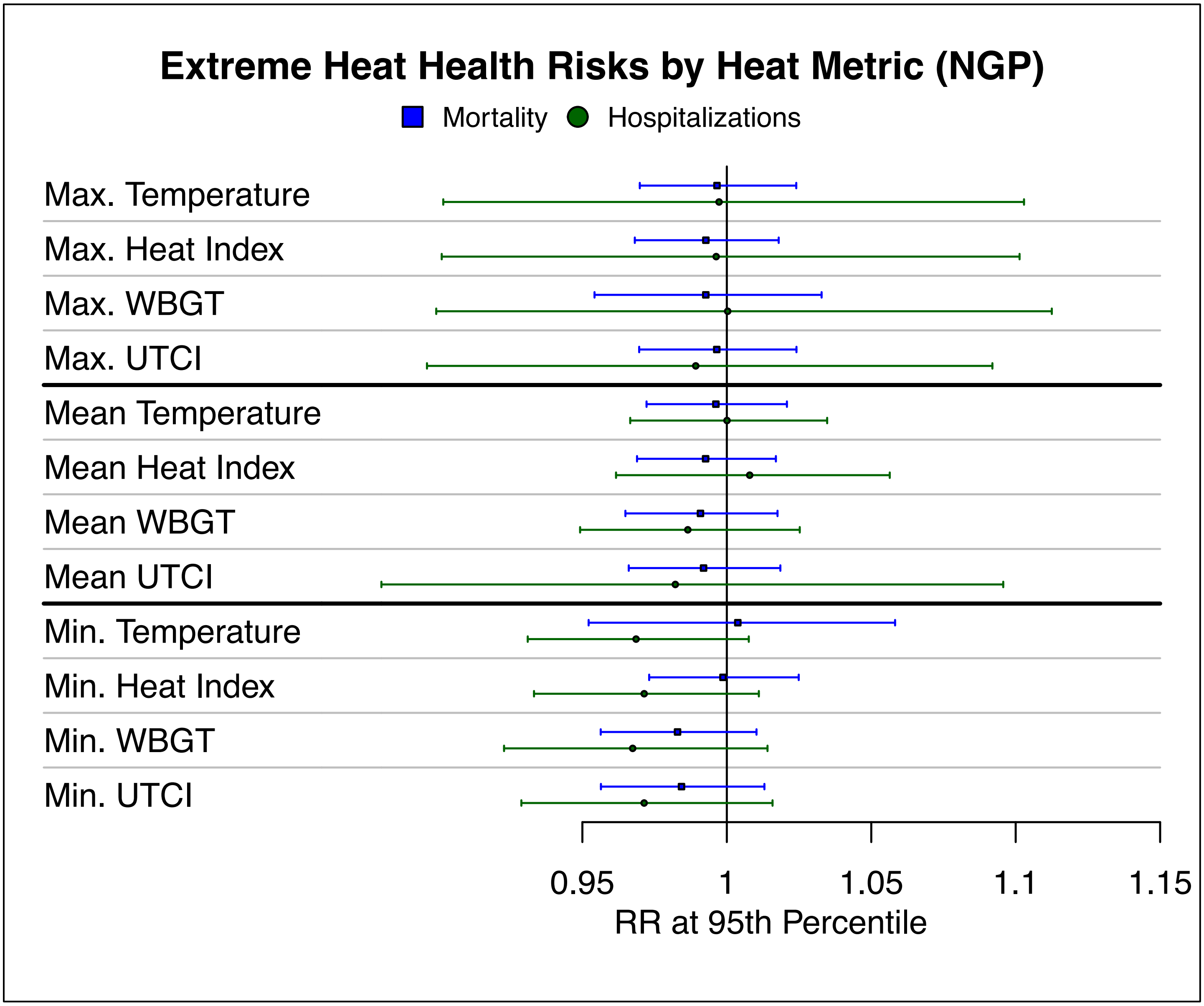

Figure S20. Southern Great Plains NCA region relative risks (RR) of mortality (blue lines with squares) and heat-associated hospitalizations (green lines with circles) at the **95th percentile** of different heat metrics in the US Medicare population, 2006-2016. Abbreviations: "Max." = maximum and "Min." = minimum. Relative risks are for the 99th percentile of each heat metric compared to the corresponding heat metric of minimum morbidity/mortality.

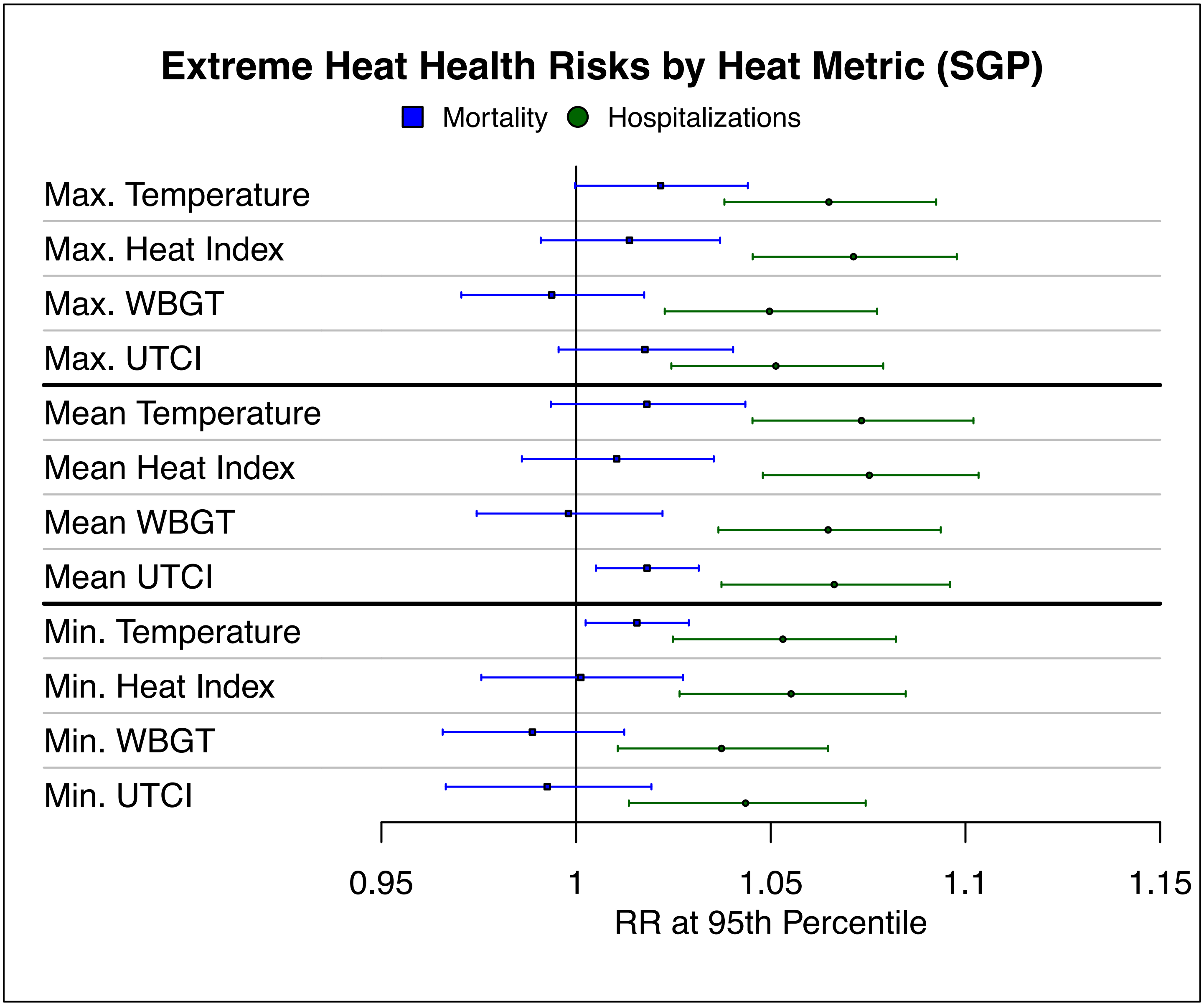

Figure S21. Southwest NCA region relative risks (RR) of mortality (blue lines with squares) and heat-associated hospitalizations (green lines with circles) at the **95th percentile** of different heat metrics in the US Medicare population, 2006-2016. Abbreviations: "Max." = maximum and "Min." = minimum. Relative risks are for the 99th percentile of each heat metric compared to the corresponding heat metric of minimum morbidity/mortality.

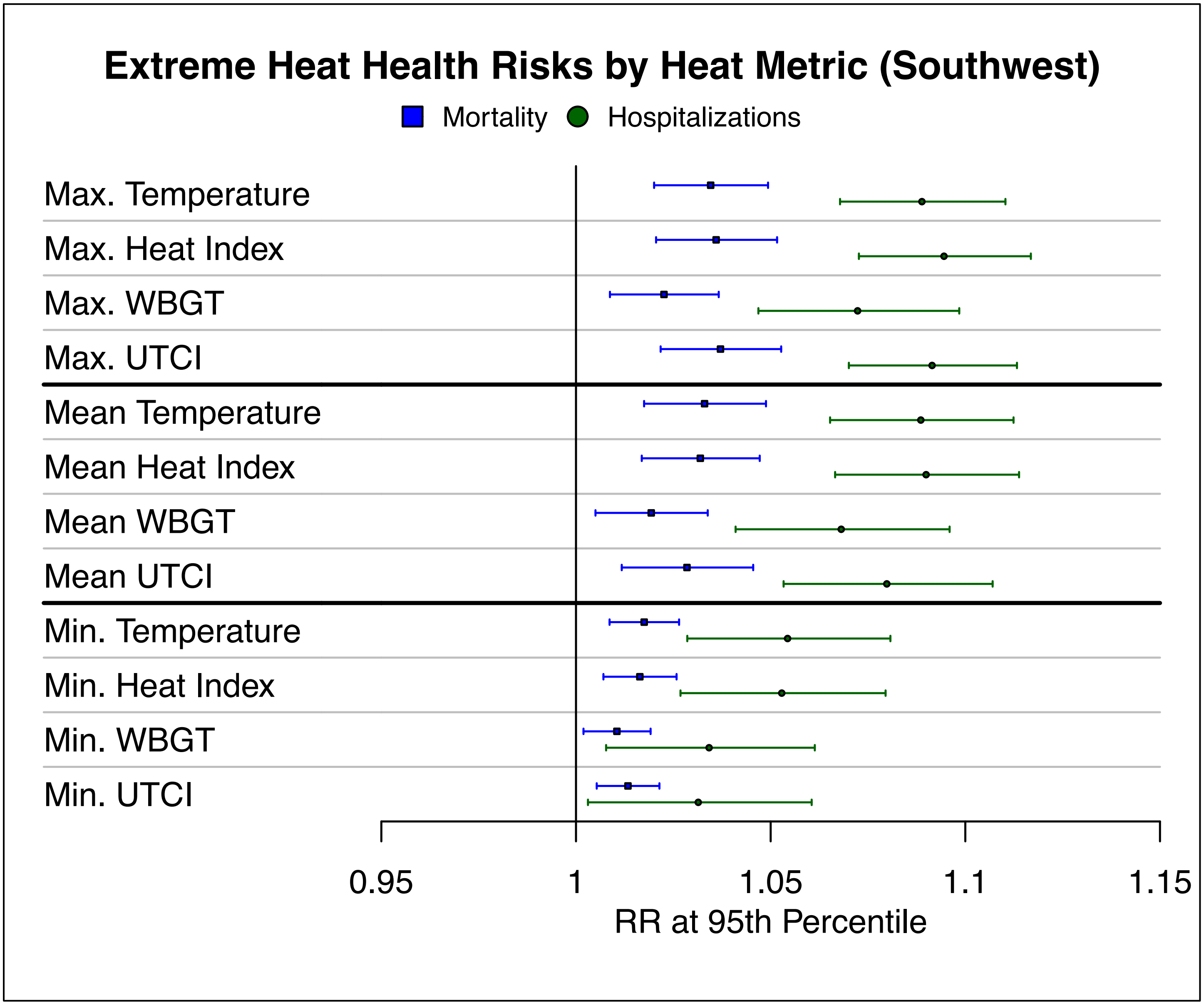

Figure S22. Northwest NCA region relative risks (RR) of mortality (blue lines with squares) and heat-associated hospitalizations (green lines with circles) at the **95th percentile** of different heat metrics in the US Medicare population, 2006-2016. Abbreviations: "Max." = maximum and "Min." = minimum. Relative risks are for the 99th percentile of each heat metric compared to the corresponding heat metric of minimum morbidity/mortality.

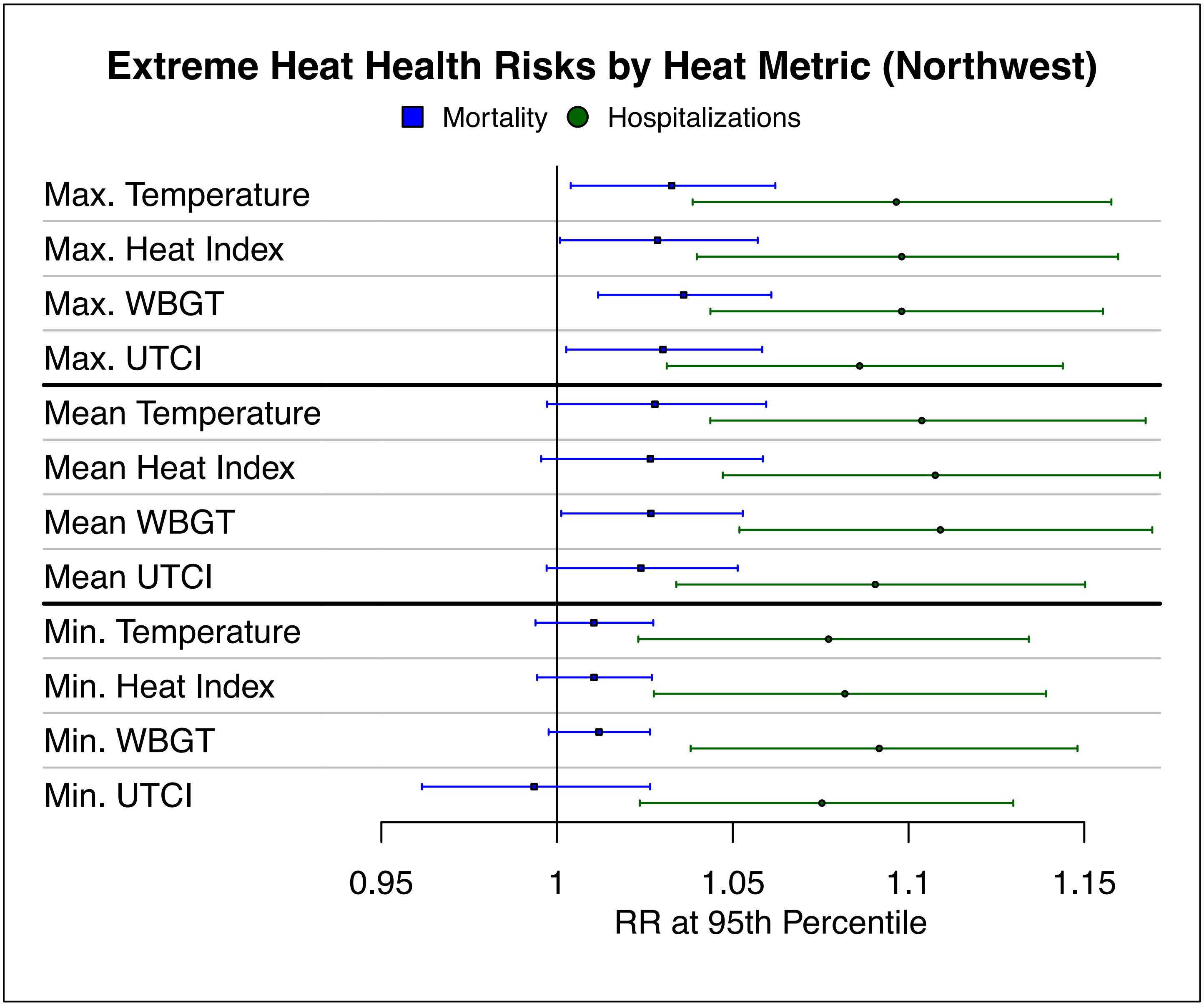

Supplement: Supplementary file 1 [file ee9-7-e261-s001.pdf]
